# Machine learning identifies prominent factors associated with cardiovascular disease: findings from two million adults in the Kashgar Prospective Cohort Study (KPCS)

**DOI:** 10.1186/s41256-022-00282-y

**Published:** 2022-12-06

**Authors:** Jia-Xin Li, Li Li, Xuemei Zhong, Shu-Jun Fan, Tao Cen, Jianquan Wang, Chuanjiang He, Zhoubin Zhang, Ya-Na Luo, Xiao-Xuan Liu, Li-Xin Hu, Yi-Dan Zhang, Hui-Ling Qiu, Guang-Hui Dong, Xiao-Guang Zou, Bo-Yi Yang

**Affiliations:** 1 Guangdong Provincial Engineering Technology Research Center of Environmental Pollution and Health Risk Assessment, Department of Occupational and Environmental Health, School of Public Health, Sun Yat-Sen University, 74 Zhongshan 2nd Road, Yuexiu District, Guangzhou, 510080 China; 2 Department of Respiratory and Critical Care Medicine, The First People’s Hospital of Kashi (The Affiliated Kashi Hospital of Sun Yat-Sen University), No.66, Yingbin Avenue, Kashgar City, 844000 China; 3 Guangzhou Center for Disease Control and Prevention, Guangzhou, 510440 China; 4 Department of Research and Development, Nanfang Hospital, Southern Medical University, Guangzhou, 510515 China

**Keywords:** Cardiovascular disease (CVD), Prediction, Prominent factors, Machine learning, Kashgar prefecture

## Abstract

**Background:**

Identifying factors associated with cardiovascular disease (CVD) is critical for its prevention, but this topic is scarcely investigated in Kashgar prefecture, Xinjiang, northwestern China. We thus explored the CVD epidemiology and identified prominent factors associated with CVD in this region.

**Methods:**

A total of 1,887,710 adults at baseline (in 2017) of the Kashgar Prospective Cohort Study were included in the analysis. Sixteen candidate factors, including seven demographic factors, 4 lifestyle factors, and 5 clinical factors, were collected from a questionnaire and health examination records. CVD was defined according to International Clinical Diagnosis (ICD-10) codes. We first used logistic regression models to investigate the association between each of the candidate factors and CVD. Then, we employed 3 machine learning methods—Random Forest, Random Ferns, and Extreme Gradient Boosting—to rank and identify prominent factors associated with CVD. Stratification analyses by sex, ethnicity, education level, economic status, and residential setting were also performed to test the consistency of the ranking.

**Results:**

The prevalence of CVD in Kashgar prefecture was 8.1%. All the 16 candidate factors were confirmed to be significantly associated with CVD (odds ratios ranged from 1.03 to 2.99, all *p* values < 0.05) in logistic regression models. Further machine learning-based analysis suggested that age, occupation, hypertension, exercise frequency, and dietary pattern were the five most prominent factors associated with CVD. The ranking of relative importance for prominent factors in stratification analyses showed that the factor importance generally followed the same pattern as that in the overall sample.

**Conclusions:**

CVD is a major public health concern in Kashgar prefecture. Age, occupation, hypertension, exercise frequency, and dietary pattern might be the prominent factors associated with CVD in this region.In the future, these factors should be given priority in preventing CVD in future.

**Supplementary Information:**

The online version contains supplementary material available at 10.1186/s41256-022-00282-y.

## Introduction

Cardiovascular disease (CVD) is a leading cause of morbidity and mortality worldwide, and its case number has increased from 271 million in 1990 to 523 million in 2019 [1]. China is also severely threatened by CVD where it is estimated that two in five deaths in China can be attributed to the disease [2]. Effective prevention and control strategies are therefore needed to reverse the rising tide of CVD, of which identification of modifiable risk factors is critical. Actually, numerous prior studies have found some common determinants, including elder age, tobacco smoking, overweight or obesity, diabetes, hypertension, and dyslipidemia [2, 3]. Also, several clinical risk prediction models have been established to estimate CVD risk by incorporating these factors [e.g., Framingham Risk Score, QRISK, and Prediction for Atherosclerotic Cardiovascular Disease Risk in China (China-PAR equations)] [4–6]. However, due to heterogeneity in the population’s characteristics, lifestyles, health status, and genetic backgrounds, epidemiological features for CVD are population-specific. For example, North China was particularly affected by obesity and high blood pressure, whereas South China was mainly affected by staple food intake and physical inactivity [7]. In China, studies estimating the CVD burden and its determinants were mainly performed in densely-populated central and eastern regions, yet in northwestern China, such evidence is still scarce.

Kashgar prefecture is located in the western part of China, and approximately 92% of its residents were of Uyghur ethnicity. The region has specific dietary habits (e.g., consuming more meat and greasy and salty food, but fewer vegetables) [5], low socioeconomic status, low health awareness, and limited availability of health services [8]. In addition, the prevalence of some CVD-related diseases such as obesity [9], dyslipidemia [10], and hypertension [11] is always high in Xinjiang Uyghur people, and thus the prevalence of CVD in this region could also be assumed to be high. However, the epidemiology of CVD in this region is scarcely reported. In addition, considering different lifestyles, environmental exposures, and genetic backgrounds, CVD-associated factors for Kashgar people may also differ from those for other populations (e.g., Han ethnicity in China). For example, previous studies have shown that CVD prediction tools using general factors substantially underestimated CVD risk in Uyghur women [12]. Still, no prior study has been performed to identify potential risk factors for CVD in people living in Kashgar prefecture.

Machine learning (ML) is a promising methodological approach. Compared to traditional statistical models that may be complicated due to the high dimensionality of data or the presence of confounding or correlated factors [13, 14], ML is advantageous in dealing with model complex and nonlinear relationships in high-dimensional data. [15]. On the clinical and epidemiological front, the use of ML in ranking and identifying key factors has been widely used for various health outcomes such as attention-deficit and hyperactivity disorder [16], childhood obesity [17], Covid-19 death [18], cancer mortality [19], and under-five mortality [20]. In this study, we estimated the prevalence and factors associated with CVD in population living in Kashgar prefecture by analyzing baseline data of approximately two million adults from the Kashgar Prospective Cohort Study (KPCS). In addition, we employed ML methods to rank and identify the most prominent factors associated with the disease.

## Methods

### Study population

This study analyzed baseline data of the KPCS, which is an ongoing large longitudinal study based on Free Universal Health Examination Programmes in Kashgar prefecture, Xinjiang, China. Detailed information on the protocol of the KPCS is summarized in Additional file 1: Cohort Profile. Briefly, since January 2016, the local government provided free annual screening health examinations for all residents living in Kashgar prefecture (including Kashi city and 11 surrounding counties), to facilitate health management. Each resident was assigned a questionnaire to collect data on demographics, lifestyles, and medical history. In addition, a series of medical examinations, including anthropometric measurements, physical examinations, blood and urinary tests, and imaging examinations, were performed in the local community/village health service centers by professional and trained medical teams following standard protocols. All residents were encouraged to participate in the health examination program yearly.

**Fig. 1:**
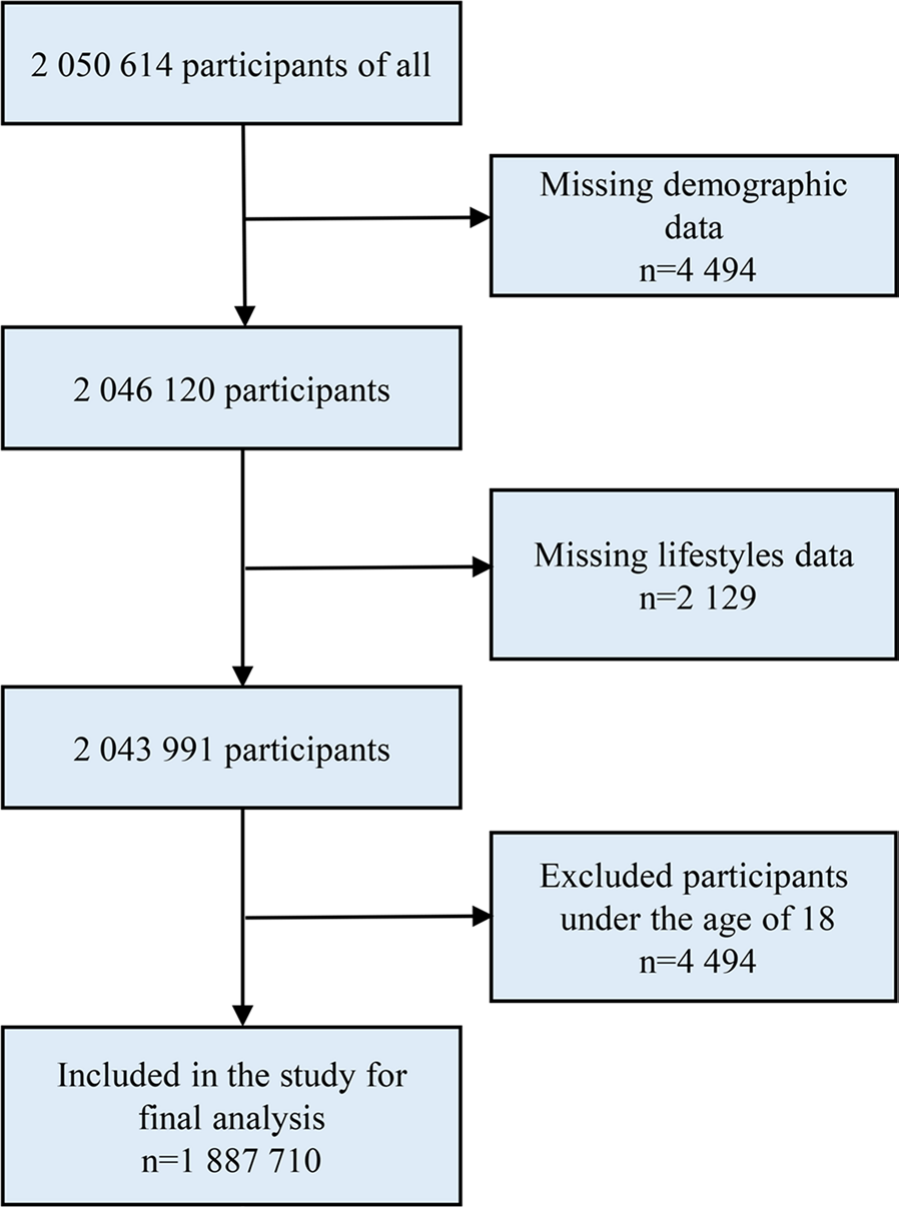
Flowchart of study participants selection

The KPCS is an open (dynamic) cohort with no ending date, and we have updated the cohort to 2020. Since the health examination in 2016 was still in its pilot period and had not been well publicized, we used participants’ data collected in 2017 as baseline data (a large proportion of residents participated in the examination since 2017). For the present study, we only analyzed the baseline data because the follow-up period is too short (4 years) to obtain enough new CVD cases for analysis. Initially, 2,050,614 individuals were included. We then excluded 4494 individuals with missing demographic data, 2129 with missing lifestyle data, and 156,281 individuals aged less than 18 years old. Finally, 1,887,710 participants were included in the analysis (Fig. 1). Each participant signed a consent form to authorize the government to derive data from medical screening. We got permission from the government to use data collected via questionnaires and health examinations, and the study protocol was approved by the Ethical Committee of the First People’s Hospital of Kashi.

### CVD diagnosis

We used the International Classification of Diseases, 10th Revision (ICD-10) to identify CVD patients from their medical records. Participants with at least one of the following codes were defined as having CVD: G45 (transient cerebral ischemic attacks and related syndromes), I05-I09 (chronic rheumatic heart disease), I20 (angina pectoris), I21-I23 (myocardial infarction), 124-I25 (other ischemic heart diseases), I50 (heart failure), I51 (complications and ill-defined descriptions of heart disease), I60 (subarachnoid hemorrhage), I61 (intracerebral hemorrhage), I62 (other nontraumatic intracranial hemorrhage), 163 (cerebral infarction), and I64 (stroke).

### Measurements of candidate factors

The choice of candidate factors associated with CVD depended on their availability in the dataset. We included factors that were previously reported to be associated with CVD, and their associations were biologically plausible [2, 21–23]. We used a questionnaire to collect data on demographic and socioeconomic factors, including age (years), sex (men, women), ethnicity (Han, Uyghur, other nationality), occupation (unemployed, worker, farmer, office clerk, others), residential setting (rural, urban), education level (years of schooling ≤ 9, years of schooling > 9), and economic status (poor household, non-poor household).

Also, we employed the questionnaire to collect data on lifestyle factors including tobacco smoking, alcohol use, dietary pattern, and physical activity. Non-smokers were defined as participants who never smoked, smokers were defined as participants who smoked during the past year or quit smoking less than 6 months, and former smokers were defined as participants who quit smoking for more than 6 months. Similarly, non-drinkers were defined as participants who never drank alcohol regularly, and ever-drinkers were defined as participants who had ever drunk alcohol at least once a week for at least six months. Dietary patterns were assessed by asking “Which dietary pattern do you adopt? omnivore, plant-heavy, or meat-heavy?” Participants were classified as “omnivore diet”, “meat-heavy diet” and “plant-heavy diet” according to their answers. Exercise frequency was determined by asking “Other than your regular work, how often did you engage in physical activity during the past 6 months” Participants were classified into four groups: no exercise, < 1 day/week; 1–6 days/week; and 7 days/week.

Body height and weight were examined according to the recommendation of the World Health Organization, and then body mass index (BMI) was calculated. Obese, overweight, and normal weight was defined as a BMI ≥ 28, 24–28, and < 24 kg/m^2^, respectively [24]. Systolic blood pressure (SBP) and diastolic blood pressure (DBP) were measured by trained and certified nurses using standardized mercuric-column sphygmomanometers. After participants were instructed to relax and sit calmly for 5 min, blood pressure measurements were taken thrice at 5 min intervals, and the average readings were recorded. We defined hypertension as SBP ≥ 140 mmHg and/or DBP ≥ 90 mmHg, and/or reported receipt of antihypertensive medications within 2 weeks before the measurement [25].

Blood samples were collected from the antecubital vein after an overnight fast. Fasting blood glucose, total cholesterol, and triglycerides were determined using an autoanalyzer (Hitachi). Diabetes was defined as a fasting glucose level of at least 126 mg/dL (or 7.0 mmol/L) and/or intaking antidiabetic medication [26]. Hypercholesterolemia was defined as total cholesterol levels ≥ 240 mg/dL (or 6.2 mmol/L), and hypertriglyceridemia as triglyceride levels ≥ 200 mg/dL (or 2.3 mmol/L) [27].

### Statistical analysis

To facilitate reading, we schematically described the procedures of data analysis in Additional file 1. Firstly, we employed logistic regression analysis to investigate the association between each of the studied factors and CVD. We fitted both crude and adjusted models (i.e., all potential factors were incorporated into the model to adjust for each other). Effects estimates were presented as odds ratios (ORs) with 95% confidence intervals (CIs). Stratification analysis was also performed to assess sex disparity and ethnic disparity in factors associated with CVD, and Student’s t-test was applied to compare ORs between subgroups. Reported *p* values are 2-sided, and a *p* value < 0.05 indicated statistical significance.

Next, we ranked and identified prominent factors associated with CVD using ML methods. For this purpose, we first randomly split data into a training set (70%; used for training models) and a test set (30%; used for testing the model performance). Then, Synthetic Minority Over-sampling Technique [28] was applied to overcome data imbalance (caused by relatively low CVD prevalence) in the training set. Then, after we used Boruta algorithm [29] to filter out irrelevant factors to CVD, three ML models—Random Forest (RF), Random Ferns (RFs), and Extreme Gradient Boosting (XGBoost)—were independently constructed and the corresponding importance scores of factors were then calculated. The above three methods are easy to train and test, which have also been demonstrated to have good performance in other studies [30, 31]. The choice of hyperparameters for each ML method was optimized on the training dataset using ten-fold cross validation. We ranked these factors based on the importance scores generated by the classification models with higher scores indicating greater importance.

Further, we assessed and compared the performance of the three ML methods in the test set using two indicators—area under the ROC curve and the area under the Precision-Recall curve [32]. Based on the best method selected, we adopted a stepwise selection procedure to identify the most prominent factors: we began by including the top rank factor and incrementally included other factors according to their rankings until we reached a minimal-optical set of factors (i.e., the parsimonious model). To identify the minimal-optical subset, we first calculated the area under the ROC (i.e., AUC value) [33] for each model that incrementally incorporated the candidate factors (i.e., the number of factors increased from 1 to 16). Then, we plotted the number of factors (x-axis) against the AUC values (y-axis), and when the AUC curve reaches a plateau, the corresponding factors formed the minimal-optical subset. Factors retained in this subset were identified as prominent factors associated with CVD. In addition, we performed stratified analysis to examine whether the ranking of prominent factors was robust in populations with different demographic factors (i.e., sex, ethnicity, education level, economic status, and residential settings).

All statistical analyses were performed using R version 4.0.5 (R Foundation for Statistical Computing). The R packages “ranger”, “rFerns”, and “xgboost” were used for training RF, RFs, and XGBoost models respectively.

### Role of the funding source

The funder had no role in the study design, data collection, analysis, interpretation of the results, or drafting of the manuscript. The corresponding authors had full access to all the study data and had full responsibility for the decision to submit it for publication.

## Results

### Basic characteristics

The mean (SD) age of the included participants was 36.7 (15.3) and nearly half of them were women (51.1%) (Table 1). About 96.0% of the participants were of Uyghur ethnicity, 75.7% lived in rural areas, 12.3% had a high-school education or higher, 28.1% were from poor households and 76.7% were farmers. A total of 153,649 participants (8.1%) were diagnosed with CVD. Compared with participants without CVD, those with CVD were more likely to be older (proportion of participants over 45 years old, 58.7% vs. 31.6%), be women (52.7% vs. 50.9%), be overweight or obese (57.3% vs. 47.7%), and have diabetes (8.9% vs. 4.0%) or hypertension (33.7% vs. 11.7%).

**Table 1:** Characteristics of study participants by CVD group

Characteristics	No. (%) of participants			*p* value
	Overall (N = 1,887,710)	Participants without CVD (N = 1,734,061)	Participants with CVD (N = 153,649)	
Sex				< 0.0001
Man	923,655 (48.9%)	850,926 (49.1%)	72,729 (47.3%)	
Woman	964,055 (51.1%)	883,135 (50.9%)	80,920 (52.7%)	
Age (year)				< 0.0001
18–45	1,249,754 (66.2%)	1,186,333 (68.4%)	63,421 (41.3%)	
45–65	476,332 (25.2%)	416,073 (24.0%)	60,259 (39.2%)	
≥ 65	161,624 (8.6%)	131,655 (7.6%)	29,969 (19.5%)	
Ethnicity				< 0.0001
Han nationality	56,078 (3.0%)	51,030 (3.0%)	5,048 (3.3%)	
Uyghur nationality	1,811,752 (96.0%)	1,665,177 (96.0%)	146,575 (95.4%)	
Other	19,880 (1.0%)	17,854 (1.0%)	2026 (1.3%)	
Residential setting				< 0.0001
Urban	457,972 (24.3%)	420,141 (24.2%)	37,831 (24.6%)	
Rural	1,429,738 (75.7%)	1,313,920 (75.8%)	115,818 (75.4%)	
Economic status				< 0.0001
Poor household^a^	531,321 (28.1%)	489,253 (28.2%)	42,068 (27.4%)	
Non-poor household	1,356,389 (71.9%)	1,244,808 (71.8%)	111,581 (72.6%)	
Education level				< 0.0001
Years of schooling > 9	232,335 (12.3%)	216,417 (12.5%)	15,918 (10.4%)	
Years of schooling ≤ 9	1,655,375 (87.7%)	1,517,644 (87.5%)	137,731 (89.6%)	
Occupation				< 0.0001
Unemployed	76,738 (4.0%)	69,099 (4.0%)	7639 (5.0%)	
Worker	105,740 (5.6%)	82,948 (4.8%)	22,792 (14.8%)	
Farmer	1,447,299 (76.7%)	1,338,807 (77.2%)	108,492 (70.6%)	
Office clerk	75,476 (4.0%)	70,528 (4.0%)	4948 (3.2%)	
Other	182,457 (9.7%)	172,679 (10.0%)	9778 (6.4%)	
Smoking status				< 0.0001
Non-smokers	1,643,712 (87.1%)	1,511,676 (87.2%)	132,036 (85.9%)	
Current smokers	209,372 (11.1%)	191,857 (11.1%)	17,515 (11.4%)	
Former smokers	34,626 (1.8%)	30,528 (1.7%)	4098 (2.7%)	
Alcohol use				< 0.0001
Non-drinkers	1,777,621 (94.2%)	1,634,865 (94.3%)	142,756 (92.9%)	
Ever-drinkers	110,089 (5.8%)	99,196 (5.7%)	10,893 (7.1%)	
Exercise frequency				< 0.0001
7 days/week	236,257 (12.5%)	223,137 (12.9%)	13,120 (8.5%)	
1–6 days/week	29,873 (1.6%)	27,927 (1.6%)	1946 (1.3%)	
< 1 day/week	61,804 (3.3%)	57,981 (3.3%)	3823 (2.5%)	
No exercise	1,559,776 (82.6%)	1,425,016 (82.2%)	134,760 (87.7%)	
Dietary pattern				< 0.0001
Omnivore diet	1,703,369 (90.2%)	1,567,719 (90.4%)	135,650 (88.3%)	
Plant-heavy diet	122,280 (6.5%)	110,104 (6.4%)	12,176 (7.9%)	
Meat-heavy diet	62,061 (3.3%)	56,238 (3.2%)	5823 (3.8%)	
Weight group				< 0.0001
Normal weight^b^	914,473 (48.4%)	907,673 (52.3%)	65,564 (42.7%)	
Overweight or obesity	973,237 (51.6%)	826,388 (47.7%)	88,085 (57.3%)	
Diabetes^c^				< 0.0001
No	1,805,071 (95.6%)	1,665,009 (96.0%)	140,062 (91.2%)	
Yes	82,639 (4.4%)	69,052 (4.0%)	13,587 (8.8%)	
Hypertension^d^				< 0.0001
No	1,632,674 (86.5%)	1,530,804 (88.3%)	101,870 (66.3%)	
Yes	255,036 (13.5%)	203,257 (11.7%)	51,779 (33.7%)	
Hypercholesterolemia^e^				< 0.0001
No	1,495,620 (79.2%)	1,382,778 (79.7%)	112,842 (73.4%)	
Yes	392,090 (20.8%)	351,283 (20.3%)	40,807 (26.6%)	
Hypertriglyceridemia^f^				< 0.0001
No	1,409,118 (74.6%)	1,303,254 (75.2%)	105,864 (68.9%)	
Yes	478,592 (25.4%)	430,807 (24.8%)	47,785 (31.1%)	

^a^Poor household was defined as a net annual household income ≤ 2952 Yuan per person

^b^Normal weight including underweight (Body mass index < 18.5)

^c^Diabetes was defined as a fasting blood glucose of 7.0 mmol/L or higher and/or having antidiabetic therapy

^d^Hypertension was defined as mean systolic blood pressure higher than 140 mmHg, mean diastolic blood pressure higher than 90 mmHg, and/or being on antihypertensive drugs within 2 weeks

^e^Hypercholesterolemia was defined as a total cholesterol level of 240 mg/dL or higher

^f^Hypertriglyceridemia was defined as a triglyceride level of 200 mg/dL or higher

### Prevalence of CVD in different populations

We estimated high demographic and geographical heterogeneity in CVD prevalence. More specifically, the prevalence was higher in women (8.4%) than in men (7.9%) and increased with age groups (from 5.1% in 18–45 years to 18.5% in age ≥ 65 years). Participants of Uyghur ethnicity had the lowest prevalence (8.1%), and the prevalence was relatively higher in Han (9.0%) and “other” ethnicities. Participants living in urban areas (8.3%) had a comparable prevalence of CVD to those living in rural areas (8.1%) (Additional file 1: Table S1). Geographically, the highest CVD prevalence was found in Kashi City (19.8%), followed by Zepu County (18.1%), Bachu County (11.1%), Yecheng County (7.0%) and Yingjisha County had the lowest prevalence rate of 3.2% (Additional file 1: Figure S1; Table S2).

### Association between candidate factors and CVD

We estimated the associations of seven demographic factors, four lifestyle factors, and five clinical factors with CVD prevalence, and observed significant associations for all the explored factors (Table 2). More specifically, being women, older, non-Uyghur nationality, living in urban areas, non-poor household, less educated, and being workers were associated with higher odds of CVD (ORs ranged from 1.03 to 2.99, all *p* < 0.05). Compared with the unemployed participants, being farmers, office clerks, and taking other jobs (except for being workers) had lower odds of CVD (ORs ranged from 0.62 to 0.78, all *p* < 0.0001). Of the four behavioral factors, participants who were smokers or former smokers, ever-drinkers, physical inactivity, and adopted meat-heavy or plant-heavy dietary patterns had higher odds of CVD (ORs ranged from 1.06 to 1.60, all *p* < 0.05). Clinical factors including overweight or obesity, diabetes, hypertension, hypercholesterolemia, and hypertriglyceridemia (ORs ranged from 1.07 to 2.59, all *p* < 0.0001) were also associated with higher odds of CVD (Table 2). In stratified analysis, the above associations in subgroup populations were generally consistent with those in the overall population (Additional file 1: Table S3).

**Table 2:** Association between CVD and candidate factors in unadjusted models and adjusted model

Factors	Unadjusted model (n = 1,887,710)		Adjusted model^a^ (n = 1,887,710)	
	OR (95% CI)	*p* value	OR (95% CI)	*p* value
*Sex*				
Man	1 (Reference)		1 (Reference)	
Woman	1.07 (1.06–1.08)	< 0.0001	1.15 (1.14–1.16)	< 0.0001
*Age (year)*				
18–45	1 (Reference)		1 (Reference)	
45–65	2.71 (2.68–2.74)	< 0.0001	2.01 (1.98–2.03)	< 0.0001
≥ 65	4.26 (4.20–4.32)	< 0.0001	2.99 (2.94–3.04)	< 0.0001
*Ethnicity*				
Uyghur nationality	1 (Reference)		1 (Reference)	
Han nationality	1.12 (1.09–1.16)	< 0.0001	1.03 (1.00-1.07)	0.0470
Other	1.29 (1.23–1.35)	< 0.0001	1.09 (1.03–1.15)	0.0040
*Residential setting*				
Rural	1 (Reference)		1 (Reference)	
Urban	1.02 (1.01–1.03)	0.0006	1.03 (1.02–1.05)	< 0.0001
*Economic status*				
Poor household	1 (Reference)		1 (Reference)	
Non-poor household	1.04 (1.03–1.05)	< 0.0001	1.05 (1.04–1.07)	< 0.0001
*Education level*				
Years of schooling > 9	1 (Reference)		1 (Reference)	
Years of schooling ≤ 9	1.23 (1.21–1.26)	< 0.0001	1.23 (1.21–1.26)	< 0.0001
*Occupation*				
Unemployed	1 (Reference)		1 (Reference)	
Worker	2.49 (2.42–2.56)	< 0.0001	2.81 (2.73–2.89)	< 0.0001
Farmer	0.73 (0.72–0.75)	< 0.0001	0.78 (0.76–0.80)	< 0.0001
Office clerk	0.63 (0.61–0.66)	< 0.0001	0.79 (0.76–0.82)	< 0.0001
Other	0.51 (0.50–0.53)	< 0.0001	0.62 (0.60–0.64)	< 0.0001
*Smoking status*				
Non-smokers	1 (Reference)		1 (Reference)	
Current smokers	1.05 (1.03–1.06)	< 0.0001	1.19 (1.17–1.22)	< 0.0001
Former smokers	1.54 (1.49–1.59)	< 0.0001	1.29 (1.24–1.34)	< 0.0001
*Alcohol use*				
Non-drinkers	1 (Reference)		1 (Reference)	
Ever-drinkers	1.26 (1.23–1.28)	< 0.0001	1.27 (1.24–1.30)	< 0.0001
*Exercise frequency*				
7 days/week	1 (Reference)		1 (Reference)	
1–6 days/week	1.19 (1.13–1.24)	< 0.0001	1.17 (1.11–1.23)	< 0.0001
< 1 day/week	1.12 (1.08–1.16)	< 0.0001	1.06 (1.02–1.10)	0.0030
No exercise	1.61 (1.58–1.64)	< 0.0001	1.60 (1.57–1.63)	< 0.0001
*Dietary pattern*				
Omnivore diet	1 (Reference)		1 (Reference)	
Plant-heavy diet	1.28 (1.25–1.30)	< 0.0001	1.37 (1.34–1.39)	< 0.0001
Meat-heavy diet	1.20 (1.16–1.23)	< 0.0001	1.23 (1.20–1.27)	< 0.0001
*Weight group*				
Normal weight	1 (Reference)		1 (Reference)	
Overweight or obesity	1.48 (1.46–1.49)	< 0.0001	1.08 (1.07–1.09)	< 0.0001
*Diabetes*				
No	1 (Reference)		1 (Reference)	
Yes	2.34 (2.29–2.38)	< 0.0001	1.37 (1.34–1.40)	< 0.0001
*Hypertension*				
No	1 (Reference)		1 (Reference)	
Yes	3.83 (3.78–3.87)	< 0.0001	2.59 (2.56–2.63)	< 0.0001
Hypercholesterolemia				
No	1 (Reference)		1 (Reference)	
Yes	1.42 (1.41–1.44)	< 0.0001	1.08 (1.06–1.09)	< 0.0001
*Hypertriglyceridemia*				
No	1 (Reference)		1 (Reference)	
Yes	1.37 (1.35–1.38)	< 0.0001	1.07 (1.05–1.08)	< 0.0001

*Abbreviations:* *CI:* Confidence interval; *OR:* Odds ratio

^a^All factors were incorporated into the model to adjust for each other

### Prominent factors ranked by ML methods

**Fig. 2:**
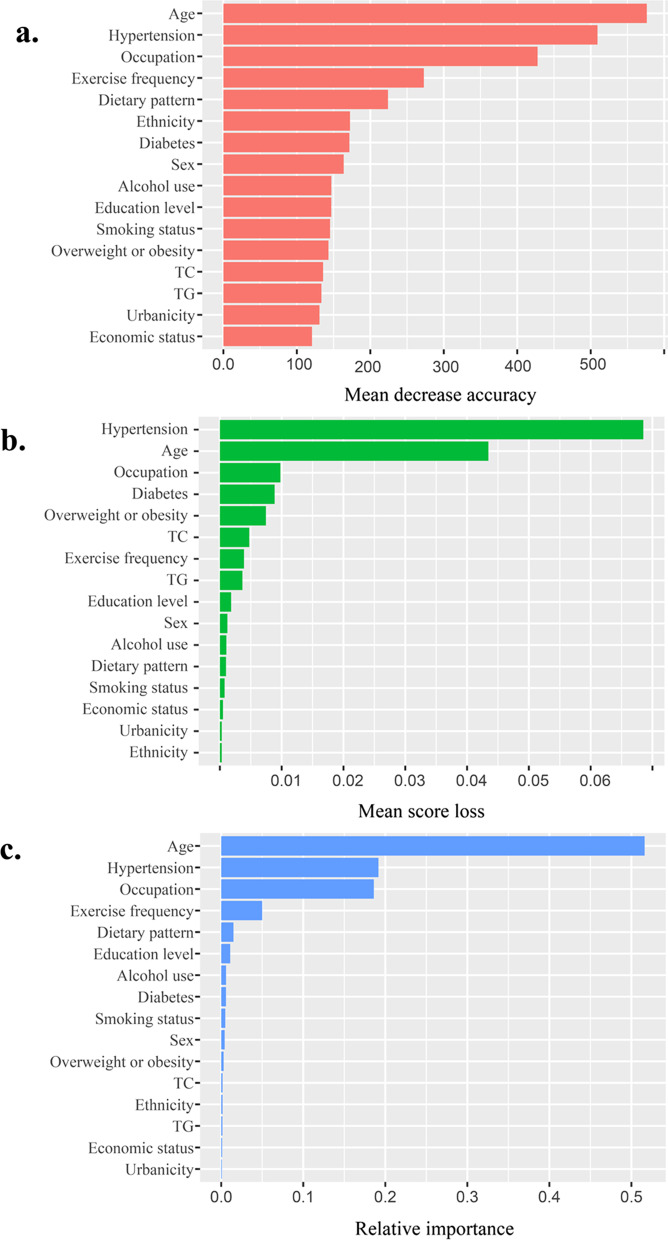
(**a**) variable importance computed from Random Forest algorithm, denoted by mean decrease accuracy; (**b**) variable importance computed from Random Ferns, denoted by mean score loss; (**c**) variable importance computed from XGBoost, denoted by relative importance

**Fig. 3:**
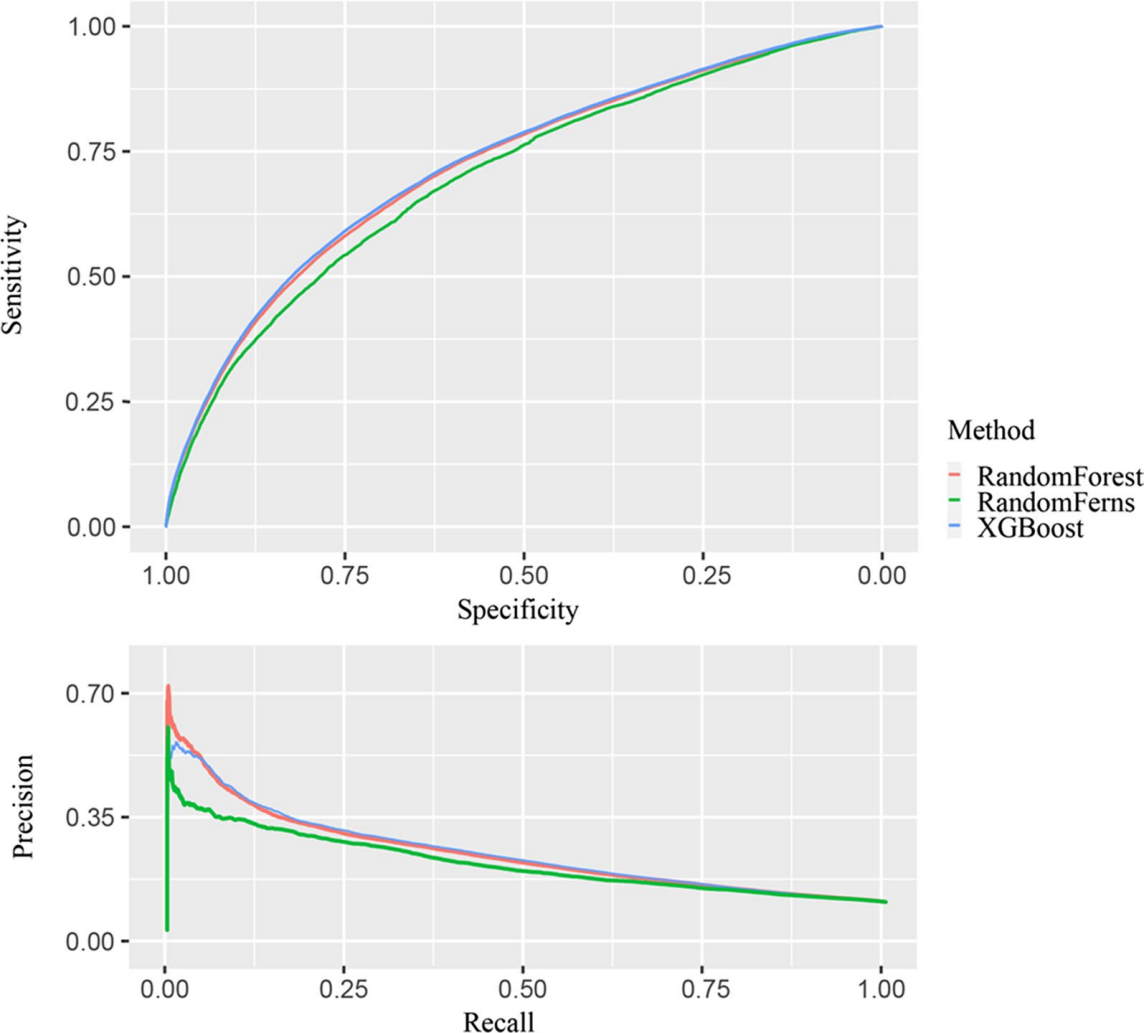
ROC curve and Precision- Recall curve for Random Forest, Random Ferns, and XGBoost models

Boruta algorithm confirmed all 16 explored factors were relevant to CVD status and were included for further analysis. (Additional file 1: Fig. S2) Within the training set, RF, RFs, and XGBoost models were established. Variable importance plot lists the factors in a descending order and the correspondent rankings generated from the three ML methods were similar (Fig. 2). Further validation analysis based on the test set showed that, RF had the highest predictive performance among the three models with an area under the ROC curve value of 0.723 (0.741 in training dataset) and an area under the PR curve value of 0.226 (0.301 in the training dataset) (Fig. 3) (Additional file 1: Table S4).

**Fig. 4:**
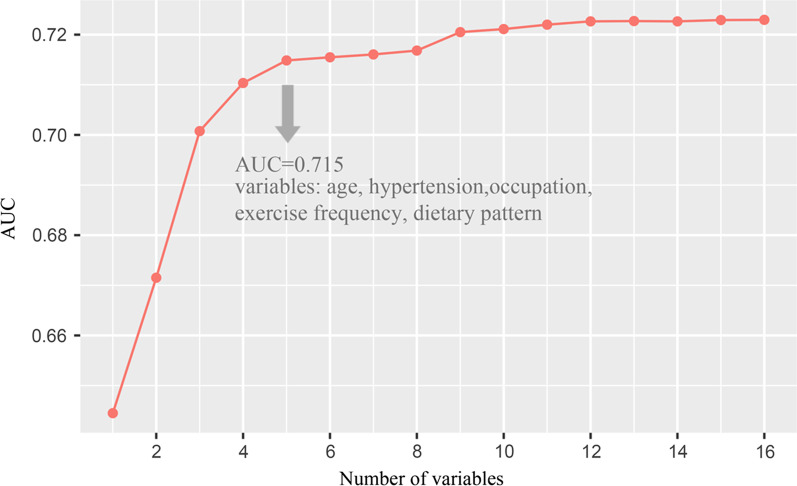
AUC values for Random Forest models in the stepwise selection procedure

In the stepwise selection procedure, we adopted the AUC curve to identify prominent factors. The AUC curve showed the model performance increased as the number of factors increased, and a plateau stage occurred when the number of factors approached five. AUC value for model with the five factors was 0.715 (AUC = 0.738 in the training dataset), which was very similar to that of the full model (i.e., all the 16 factors were included; AUC = 0.723) (Fig. 4) (Additional file 1: Table S5). The five factors were age, occupation, hypertension, exercise frequency, and dietary pattern and were thus recognized as prominent factors associated with CVD (minimal-optical set). In stratified analysis, the rankings of prominent factors in subgroups followed the same pattern as in the overall population (Additional file 1: Table S6).

## Discussion

To the best of our knowledge, this is the first investigation of CVD prevalence and factors associated with CVD in a representative sample of adults in Kashgar prefecture. We estimated that the overall prevalence of CVD was 8.1% with apparent geographic-, age-, and ethnic-specific variations. All the 16 studied demographic, lifestyle, and clinical factors were significantly associated with CVD, of which age, occupation, hypertension, exercise frequency, and dietary pattern were ranked as leading contributors to CVD by ML algorithms.

The estimated CVD prevalence in our study (8.1%) was higher than the average CVD prevalence (7.1%) across China, as reported by the Global Burden of Disease Study 2017 [34]. The result supported our original hypothesis that the CVD prevalence in Kashgar region was high because of specific lifestyles, lower SES statuses, and less healthcare resources. We were aware of only one prior study performed on Kazakh ethnicity in Kashgar prefecture, and the study reported a higher CVD prevalence than our current estimate (13.9%) [35]. A possible explanation for the discrepancy may be difference in genetic backgrounds; while the study was conducted among Kazakh population, our participants were mainly composed of Uyghur ethnicity. In addition, different lifestyles, such as dietary habits, could also be possible contributors. Collectively, evidence from the prior and our current studies indicated that CVD is highly prevalent in Kashgar prefecture, and effective intervention strategies are warranted to mitigate the burden.

Our logistic analysis showed that all the 16 explored demographic, lifestyle, and clinical factors were significantly associated with CVD. Most of the factors have been well demonstrated to be risk factors for CVD in prior studies, such as elder age, hypertension, diabetes, dyslipidemia, and tobacco smoking [3]. However, contrary to the prior evidence that being men was a CVD risk factor [2], we found women had higher odds of CVD than men. In Kashgar prefecture, men were mainly engaged in physically active jobs while women were more likely to be homemakers or engage in sedentary jobs [36]. Thus, the findings observed in our study may be explained by the difference in physical activity levels between men and women [37, 38], which is a strong protective factor for CVD [39]. We also found being workers had higher odds of CVD compared with the unemployed, which is contrary to previous findings [40]. A possible explanation for our findings may be that occupational exposures, such as chemical and physical agents, job strain, adverse job assignment, and shift rotation might have exerted hazardous effects on the cardiovascular system [41, 42]. However, due to the lack of detailed information on work types and work environments, we were unable to test the speculation.

Using ML methods, we identified that age, hypertension, occupation, dietary pattern, physical exercise were five prominent factors associated with CVD. It is difficult to directly compare our findings with the prior studies because these studies are highly heterogeneous in outcome assessment, study design, targeted population, sample size, and modeling framework. In addition, the number and types of potential factors included for modeling also varied. For example, while some studies involved hundreds of clinical factors [43, 44], the others used a dozen or so demographic and lifestyle factors [45, 46]. Despite this, key factors identified in previous studies, including age [43, 47, 48], hypertension [43, 47, 48], exercise frequency [43, 47], and dietary pattern [43, 47] were confirmed by our current study. In addition, our study identified occupation as potential important contributor to CVD, which was not generally explored in previous studies. The current findings indicate a necessity of incorporating occupational factors into CVD risk prediction and management. However, it is noteworthy that this needs to be validated by better-designed studies in the future.

We were aware of only one study aimed to develop CVD prediction model in Xinjiang. The study constructed model based on 31 factors (age, sex, smoking status, alcohol use, and 27 clinical factors) among 1508 Kazakh people living in Yili prefecture [43]. They identified age, systolic blood pressure, high-sensitivity C-reactive protein, adiponectin, and interleukin-6 as the top five factors for CVD, which is inconsistent with our findings. The discrepancy may be attributed to the difference in the number and types of factors included for prediction (i.e., while the potential factors were mostly laboratory-based clinical factors in the prior study, our current study mainly included demographic and lifestyle factors). Nevertheless, the factors we included were much easier to measure and were low-cost, thus our findings may be specifically helpful for populations who have low access to laboratory facilities but need CVD intervention.

This study has several strengths. First, the sample size is huge, which guaranteed sufficient statistical power and representativeness of the study population. Second, we used standardized protocols and instruments to perform questionnaire surveys, biological sample collection, and measurement of clinical factors in different medical centers, which ensured data homogeneity. Third, we employed ML methods to rank CVD-associated factors, which has advantages in identifying key predictors of CVD from numerous factors, since ML can review large volumes of data and discover specific trends and patterns from the data set [49].

However, this study also has limitations. First, the cross-sectional study precluded assessment of temporality, and we were thus unable to infer a causal relationship between the investigated factors and CVD. Second, demographic and lifestyle data are self-reported, thus recall bias cannot be avoided. In addition, some of these factors were measured crudely (e.g., dietary factors and alcohol use), which might have included exposure misclassification. Third, genetic factors also play partial roles in the development of CVD [50], but such data were not available in our study. Fourth, limited by the availability of variables in our dataset, the AUC of our model is not very high. Fifth, certain selection bias might exist since we recruited the participants on an entirely voluntary basis. Sixth, hospitalization and frequency of routine check-ups are also potential confounders, however, since these data were unavailable, we could not control the residual confounding. Seventh, although we provided novel and robust evidence on CVD prevention for people living in Kashgar, the generalizability of our findings to other populations is limited. However, since most of the participants were of Uyghur ethnicity, our result may be referenceable for Uyghur people living in Xinjiang province, and other populations living in neighboring regions with similar genetic backgrounds and lifestyles, especially considering the constant scarceness in this region.

## Conclusions

Our study suggests that the prevalence of CVD among Kashgar adults in Xinjiang was high and showed demographical and geographical variations. In addition, age, occupation, hypertension, exercise frequency, and dietary pattern might be the five most important factors affecting the development of CVD for people living in this area. These important factors therefore should be given priority in preventing and controlling CVD. However, given the limitations of our study, better-designed studies are needed to validate our results in the future.

## Supplementary information

**Additional file 1: Fig. S1.** Prevalence of CVD in Kashgar prefecture by city/county. **Cohort Profile.** Kashgar Prospective Cohort Study (KPCS). **Fig. S2.** Importance plot generated from Boruta algorithm. **Table S1.** Prevalence of CVD among participants with different characteristics. **Table S2.** Sample sizes and prevalence of CVD in the study area by county. **Table S3.** Associations between CVD and candidate factors stratified by sex and ethnicity. **Table S4.** Area under the receiver operating characteristic (ROC) curve and area under the precision-recall (PR) curve for RF, RFs, and XGBoost algorithms. **Table S5.** AUC values in stepwise selection procedure using RF algorithm. **Table S6.** Factor rankings for CVD computed by RF algorithm by sex, ethnicity, education level, economic status, and residential setting.

## Data Availability

The datasets used during the current study are available from the corresponding author on reasonable request.

## Abbreviations

RF: Random Forest
RFs: Random Ferns
XGBoost: Extreme Gradient Boosting
