# Supplementary material for: Machine learning identifies prominent factors associated with cardiovascular disease: findings from two million adults in the Kashgar Prospective Cohort Study (KPCS)
Source: Glob Health Res Policy. 2022 Dec 6;7:48. doi: 10.1186/s41256-022-00282-y (PMC9724436; doi:10.1186/s41256-022-00282-y)
Supplement: Supplementary file 1 — Additional file 1: Fig. S1. Prevalence of CVD in Kashgar prefecture by city/county. Cohort Profile. Kashgar Prospective Cohort Study (KPCS). Fig. S2. Importance plot generated from Boruta algorithm. Table S1. Prevalence of CVD among participants with different characteristics. Table S2. Sample sizes and prevalence of CVD in the study area by county. Table S3. Associations between CVD and candidate factors stratified by sex and ethnicity. Table S4. Area under the receiver operating characteristic (ROC) curve and area under the precision-recall (PR) curve for RF, RFs, and XGBoost algorithms. Table S5. AUC values in stepwise selection procedure using RF algorithm. Table S6. Factor rankings for CVD computed by RF algorithm by sex, ethnicity, education level, economic status, and residential setting. [file 41256_2022_282_MOESM1_ESM.docx]

**Additional File 1.**

**Machine Learning Identifies Prominent Factors Associated with Cardiovascular Disease: Findings from Two Million Adults in the Kashgar Prospective Cohort Study (KPCS)**

Jia-Xin Li, Li Li, Xuemei Zhong, Shu-Jun Fan, Tao Cen, Jianquan Wang, Chuanjiang He, Zhoubin Zhang, Ya-Na Luo, Xiao-Xuan Liu, Li-Xin Hu, Yi-Dan Zhang, Hui-Ling Qiu, Guang-Hui Dong, Xiao-Guang Zou, Bo-Yi Yang

**Table of contents**

**Figure S1. Prevalence of CVD in Kashgar prefecture by city/county.** 2

**Cohort Profile: Kashgar Prospective Cohort Study (KPCS)** 3

**Supplemental methods** 6

**Figure S2: Importance plot generated from Boruta algorithm.**. 10

**Table S1: Prevalence of CVD among participants with different characteristics** 11

**Table S2: Sample sizes and prevalence of CVD in the study area by county** 13

**Table S3: Associations between CVD and candidate factors stratified by sex and ethnicity** 16

**Table S4: Area under the receiver operating characteristic (ROC) curve and area under the precision-recall (PR) curve for RF, RFs, and XGBoost algorithms** 18

**Table S5:** **AUC values in stepwise selection procedure using RF algorithm** 19

**Table S6: Factor rankings for CVD computed by RF algorithm by sex, ethnicity, education level, economic status, and residential setting** 20

**
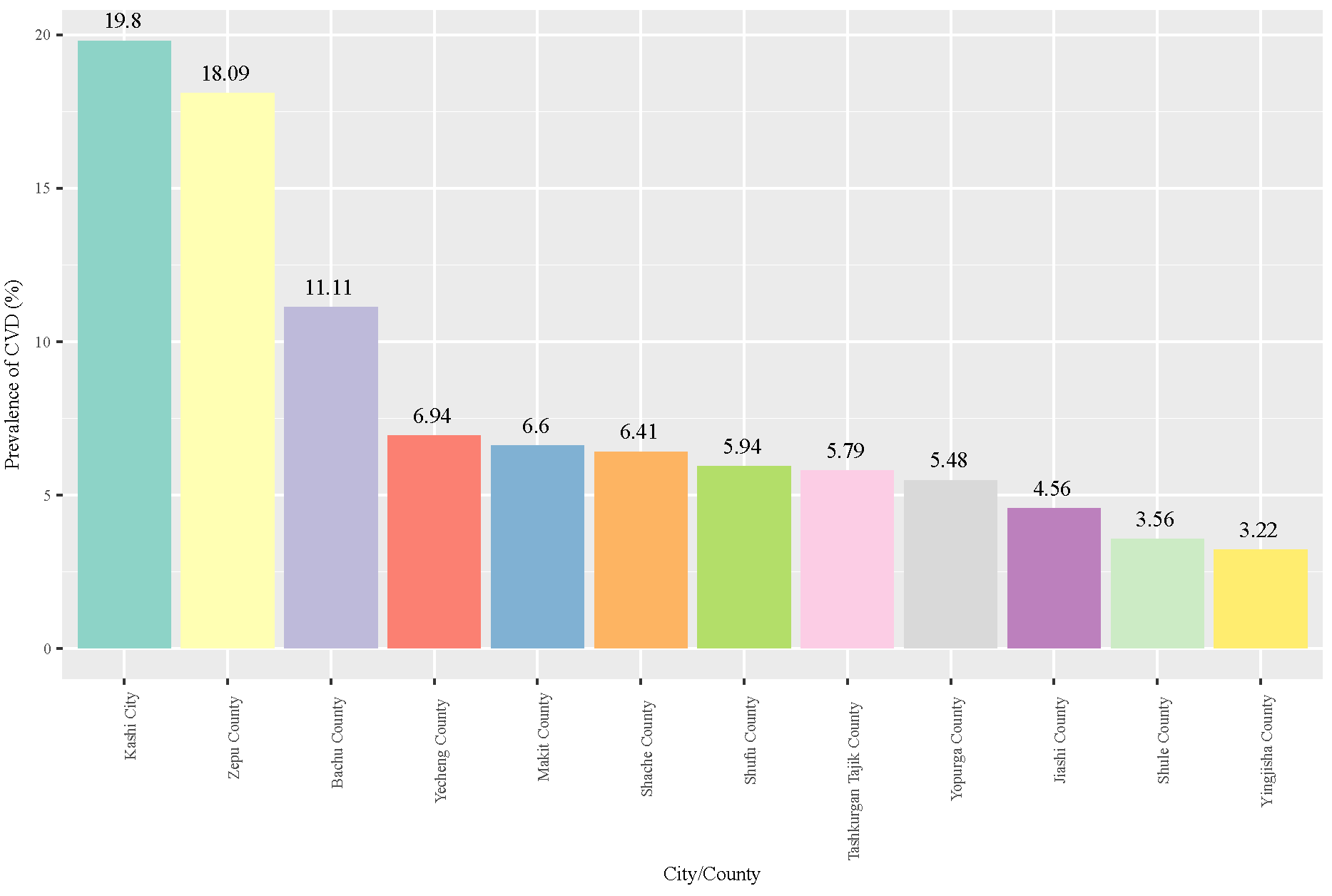
**

**Figure S1. Prevalence of CVD in Kashgar prefecture by city/county.**

**Cohort Profile: Kashgar Prospective Cohort Study (KPCS)**

**1. Brief introduction**

The Kashgar Prospective Cohort Study (KPCS) is a cohort of Kashgar residents who participated in the national health examination Program in 2017. The purpose of this cohort is to offer a wide range of health information in Kashgar prefecture for disease control and prevention.

**2. Why was the cohort setup**

Since 2016, the government of Xinjiang Uyghur Autonomous Region has provided national health screening program annually for Xinjiang residents to improve the general health status among residents. In the infancy of the project, efforts had made to modify implementation details and strengthen management, thus improving the program quality. In the year 2017, 74.1% [12] of Xinjiang residents established health screening records.

Kashgar prefecture, located in Southwestern Xinjiang, is the westernmost region in China bordering eight countries (e.g., Tajikistan, Afghanistan, Pakistan, and India). It is a settlement of Uyghur people including Kashi City and 11 counties [13]. Surrounded by desertified land, desertification in this area is more severe than in other part of Xinjiang, suffering from drought and little rainfall all year round. Besides, local people are fond of Greasy and salty foods, having a low intake of vegetables and fruits [14]. The unique geographical, ethnic, and cultural factors in Kashgar prefecture make it distinct from other parts of Xinjiang. However, few epidemiology studies were conducted in this region, and the epidemiology feature of many infections and non-communicable diseases is mostly unknown. KPCS was thus built as a cohort study in this region using data from the national health examination program, investigating a wide range of health issues.

**3. Who is in the cohort**

According to the *Implementation Plan of National Health Examination* [15], people eligible for the screening program include registered permanent residents and migrant populations living in Xinjiang province. The migrant populations refer to those who have lived in the local area ≥6 months and had obtained residence permits, except for the corps. Thus, the study population comprised all residents who have lived in Kashgar prefecture (including Kash city and 11 counties) for more than six months and underwent the health screening program between January 2017 and December 2017. The cohort included 2,050,614 eligible screening participants.

**4. How often have they been followed up**

KPCS extracted screening information of health screening program in 2017 as baseline data and was designed to follow up through data routinely collected in health screening examinations annually. The program provides free health examinations to Xinjiang residents in one-year cycles. From 2017 through 2020, 4 cycles have been conducted and the program is still ongoing. The cohort will be maintained and updated by the health screening program.

**5. What has been measured**

The variables of the KPCS were constructed from data of the health screening program. Data are collected using a standard health check-up form recommended in the *National Basic Public Health Service Specifications (Third Edition)* [16]. The KPCS database included information about:

(1) Demographic information (age, sex, occupation, living area, family address, economic status, education level, and ethnicity);

(2) Lifestyle information (physical activity, alcohol use, dietary pattern, and tobacco smoking);

(3) General conditions (body temperature, pulse rate, respiratory rate, blood pressure, height, weight, waist circumference, self-assessment of health status, self-assessment of self-care skills, cognitive function, emotional state);

(4) Physical check-up (lips, dentition, pharynx, vision, hearing, motor function, skin, iris, fundus, lymph nodes, lungs, heart, abdomen, lower extremities, dorsal foot arteries, anus, mammary glands, genitals);

(5) Existing symptoms (dizziness, headache, palpitations, chest tightness, chest pain, chronic cough, sputum, dyspnea, polydipsia, polyuria, weight loss, fatigue, joint swelling and pain, blurred vision, numbness in hands and feet, urgent urination, painful urination, constipation, diarrhea, nausea and vomiting, dizziness, tinnitus, breast tenderness, etc);

(6) Medical history (history of Cerebrovascular diseases, kidney diseases, heart diseases, vascular diseases, eye diseases, nervous system diseases, and others);

(7) Occupational hazard factors (dust, radioactive substances, physical factors, chemicals, and others);

(8) laboratory measurements **(**blood routine test, urine routine test, fasting blood glucose, urine microalbumin, fecal occult blood, glycosylated hemoglobin, hepatitis B surface antigen, liver function test, renal function test, blood lipids, cervical smear test);

(9) Auxiliary examination (electrocardiogram, abdominal ultrasound, chest x-ray);

(10) Records of inpatient and outpatient (hospitalizations in the last year);

(11) Prescription records (for patients with chronic disease, we collect information about their medication name/usage/dosage and treatment duration in the last year);

(12) History of prophylactic inoculation (we collect the name, date, and institution of vaccinations administered in the last year).

**6. What measures have been taken to control data quality**

The *Implementation Plan of National Health Examination* [15] has stipulated the details of operational requirements and procedures for this physical examination. Briefly, Township health centers and the community health service centers take responsibility for this physical examination work. Each health center is equipped with professional personnel in body inspection, imaging, laboratory test, and public health. Besides, qualified medical equipment such as color ultrasound machines, automatic biochemical analysis instruments, and DR machines are guaranteed by local medical teams. In addition, for residents living in remote villages and mountainous areas, qualified on-site physical examination services are provided. Besides, Training courses are held for quality control before every cycle of physical examination. Participants are encouraged to visit every year and the same questionnaire is filled up and the same measurements are re-examined.

**Supplemental methods**

Procedures of data analyses are shown below.


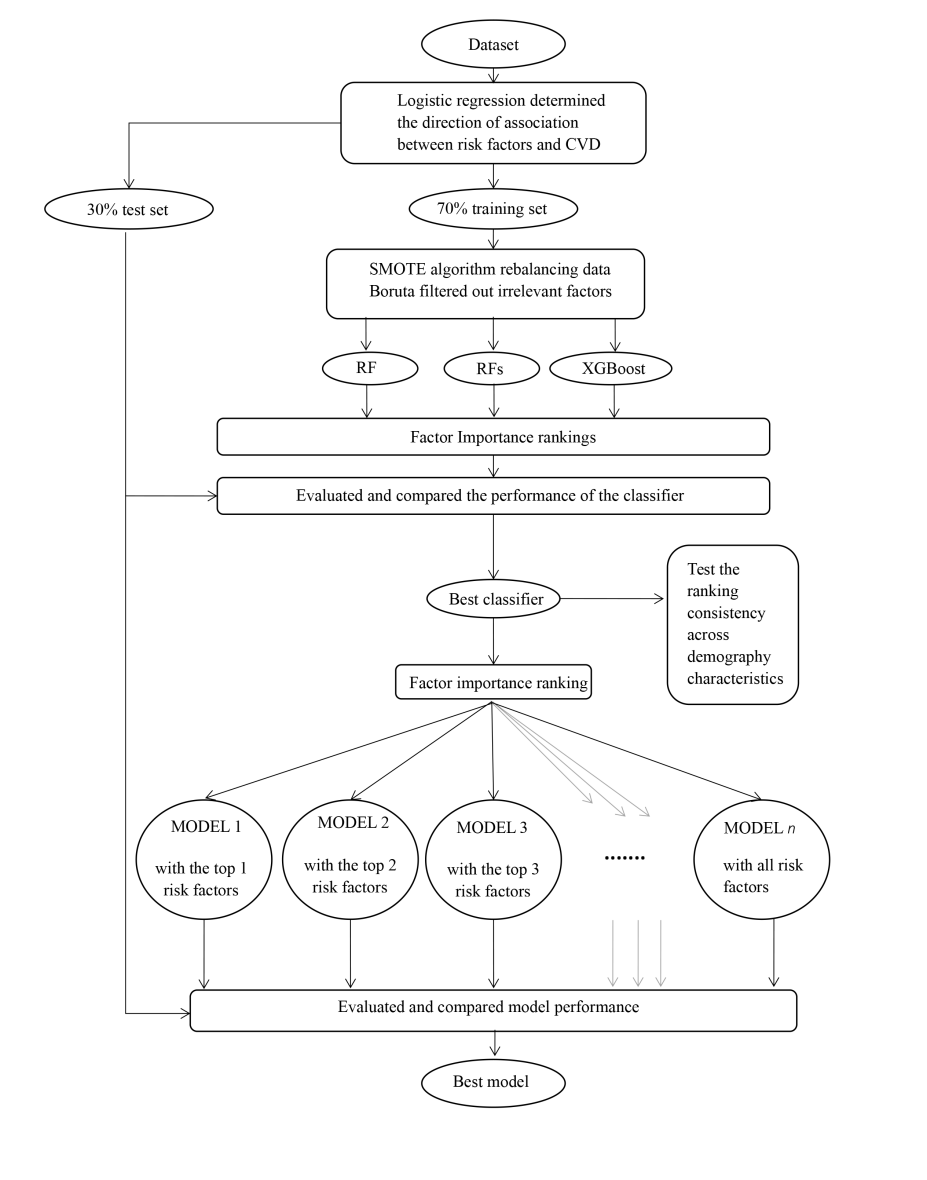


**Before model building**

First, we applied logistic regression to determine the direction of the associations between candidate factors and CVD because the following machine learning analyses cannot explain the directions between explored factors and the outcome. Then, we split data into 2 datasets randomly: a training set and a validation set (7:3). The training set was used for training models and the test set was used for testing the model performance. Since the number of people with and without CVD is highly imbalanced in our training set (8.1% of participants had CVD), which may impair our model performance, we applied the SMOTE algorithm [1] to balance our data for higher classification ability and better reliability. To filter out irrelevant factors for CVD, we also applied Boruta algorithm (a novel algorithm capable of identifying all relevant factors for the outcome) [2] to test if the included 16 factors are significant factors that discriminate CVD status before model building. Boruta is a novel algorithm capable of identifying all relevant factors for the outcome.

**Model building and factor ranking**

Factor ranking is a kind of factor selection process that ranks the factors based on their relevance and importance concerning the outcome [3]. For some classification models, the importance calculation will be performed during the model training process according to some established rules (Built-in or self-designed methods) and output importance scores of factors with the trained model.

In this study, we used three classification methods to rank factors associated with CVD. Considering the computational cost and model performance, Random Forest, Random Ferns, and Extreme Gradient Boosting were selected because they are easy to train yet have a better performance [4,5]. The Random Forest algorithm has two built-in importance measures, “Gini importance” and “Mean decrease accuracy”. The drawback of “Gini importance” is a tendency to prefer (select as important) numerical features and categorical features with high cardinality [6], which can lead to wrong conclusions. Here we selected “Mean decrease accuracy” as importance measure, in that way the importance scores of a factor can be obtained as the loss of accuracy of classification caused by the random permutation of factor values between objects. The Random Ferns algorithm has a similar importance measure as the Random Forest algorithm, but it replaced the difference in accuracy with the mean difference of the scores of the correct class, in this way extracting importance information even from objects that are misclassified [7]. The Extreme Gradient Boosting algorithm has three ways to count out the importance, namely “gain”, “frequency”, and “cover” [8]. Gain is the main reference measure of the importance for a factor in model building. Therefore, the importance measure is set by “gain” in this study. The importance scores of factors outputted from Random Forest, Random Ferns and Extreme Gradient Boosting were “Mean decrease accuracy”, “Mean score loss” and “Relative importance” respectively.

We used 10-fold cross-validation on the training set to select the hyperparameters that maximized the cross-validation error for each method. In the 10-fold cross-validation, the training data is split into 10 equally sized folds (subsets) and the models are trained on 9 folds and tested on the remaining fold. The following table shows the hyperparameters used by each model.

Table. Hyperparameters for each algorithm.

| Random Forest | Random Ferns | XGBoost |
| --- | --- | --- |
| num.trees=400  mtry=4  importance= permutation  min.node.size=1  max.depth=0 | Depth=4  ferns=500  importance=shadow  threads=0 | Booster=gbtree  max_depth=6  eta=0.1  colsample_bytree=0.8  subsample=1 |

Note: If a hyperparameter is not indicated in the table, then the default value in R packages was used. Each hyperparameter was chosen independently to reduce the risk of overfitting.

**Selection of the best classifier**

Model performance reflects the reliability of the ranking. It was evaluated on a separate test set and assessed by area under the receiver operator characteristic (ROC) curve [9] and precision-recall (PR) curve [10]. The PR curve shows precision values for corresponding sensitivity (recall) values. The closer the area under the ROC curve and PR curve to 1, the better the model performance.

**Stepwise selection procedure**

Based on the best classifier, stepwise selection procedures were used for identification of key factors: we began by including the top rank factor and incrementally included other factors according to their rankings until we reached a minimal-optical set of factors (i.e., the parsimonious model). To identify the minimal-optical subset, we first calculated the area under the ROC (i.e., AUC value). AUC is widely used for model validating and can be applied to compare model performance with existing literature [11]. The number of factors is on the horizontal axis, and the AUC value is on the vertical axis. The minimal-optical subset was approached when the AUC curve reach the plateau. Factors retained in the minimal-optical subset were identified as prominent factors of CVD. For sensitivity analyses. we test the ranking consistency stratified by sex, ethnicity, residential setting, education level, and economic status using the classification method with the highest performance we had explored.


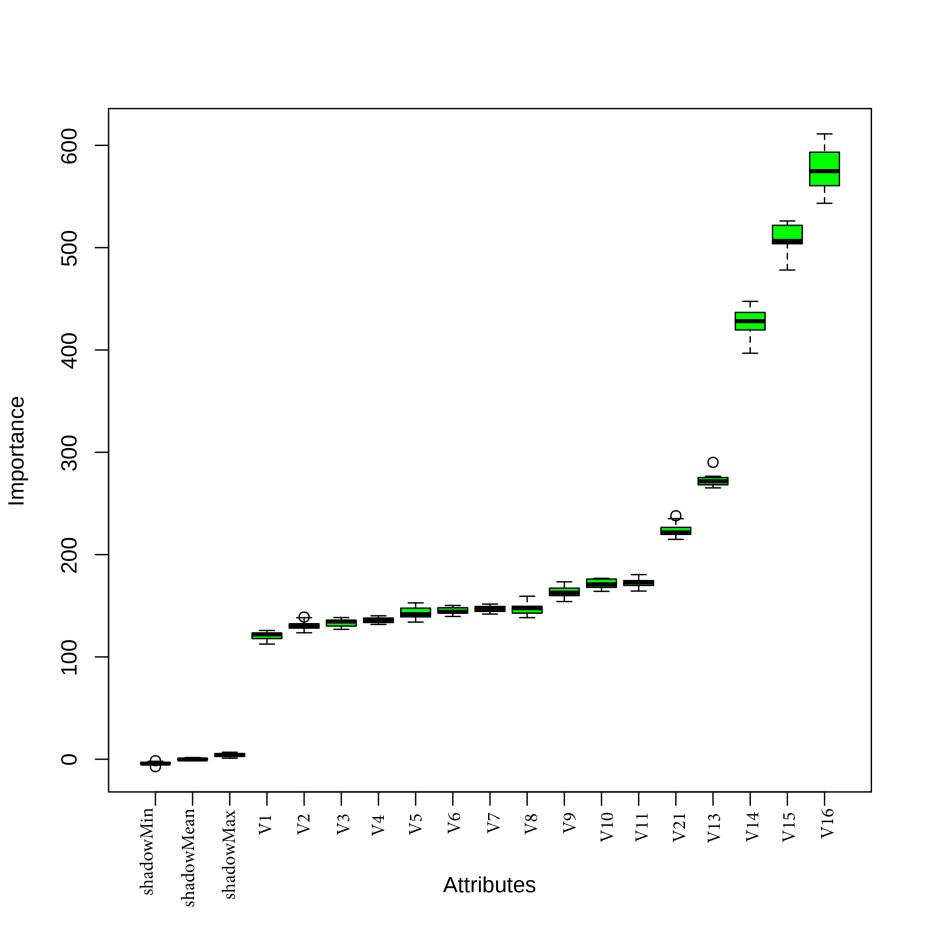


**Figure S2: Importance plot generated from Boruta algorithm.** V1: economic status; V2: urbanicity; V3:TG, V4: TC; V5: overweight or obesity; V6: smoking status; V7: education level; V8: alcohol use; V9: sex; V10: diabetes; V11: ethnicity; V12: dietary pattern; V13: exercise frequency; V14: occupation; V15: hypertension; V16: age. In Figure S2., all attributes (factors) had an importance score greater that the shadow, indicating all the 16 factors were relevant to the CVD outcome.

**Table S1: Prevalence of CVD among participants with different characteristics**

| **Characteristics** | | **No.** | | **No. (%) of people with CVD** | | ***p* value** |
| --- | --- | --- | --- | --- | --- | --- |
| Overall | | 1 887 710 |  | 153 649 (8.1%) |  |  |
| Sex | |  |  |  |  | <0.0001 |
|  | Man | 923 655 |  | 72 729 (7.9%) |  |  |
|  | Woman | 964 055 |  | 80 920 (8.4%) |  |  |
| Age (year) | |  |  |  |  | <0.0001 |
|  | 18-45 | 1 249 754 |  | 63 421 (5.1%) |  |  |
|  | 45-65 | 476 332 |  | 60 259 (12.7%) |  |  |
|  | ≥65 | 161 624 |  | 29 969 (18.5%) |  |  |
| Ethnicity | |  |  |  |  | <0.0001 |
|  | Han nationality | 56 078 |  | 5 048 (9.0%) |  |  |
|  | Uyghur nationality | 1 811 752 |  | 146 575 (8.1%) |  |  |
|  | Other | 19 880 |  | 2 026 (10.2%) |  |  |
| Residential setting | |  |  |  |  | <0.0001 |
|  | Urban | 457 972 |  | 37 831 (8.3%) |  |  |
|  | Rural | 1 429 738 |  | 115 818 (8.1%) |  |  |
| Economic status | |  |  |  |  | <0.0001 |
|  | Poor household | 531 321 |  | 42 068 (7.9%) |  |  |
|  | Non-poor household | 1 356 389 |  | 111 581 (8.2%) |  |  |
| Education level | |  |  |  |  | <0.0001 |
|  | Years of schooling >9 | 232 335 |  | 15 918 (6.9%) |  |  |
|  | Years of schooling ≤9 | 1 655 375 |  | 137 731 (8.3%) |  |  |
| Occupation | |  |  |  |  | <0.0001 |
|  | Unemployed | 76 738 |  | 7 639 (10.0%) |  |  |
|  | Worker | 105 740 |  | 22 792 (21.6%) |  |  |
|  | Farmer | 1 447 299 |  | 108 492 (7.5%) |  |  |
|  | Office clerk | 75 476 |  | 4 948 (6.6%) |  |  |
|  | Other | 182 457 |  | 9 778 (5.4%) |  |  |
| Smoking status | |  |  |  |  | <0.0001 |
|  | Non-smokers | 1 643 712 |  | 132 036 (8.0%) |  |  |
|  | Current Smokers | 209 372 |  | 17 515 (8.4%) |  |  |
|  | Former smokers | 34 626 |  | 4 098 (11.8%) |  |  |
| Alcohol use | |  |  |  |  | <0.0001 |
|  | Non-drinkers | 1 777 621 |  | 142 756 (8.0%) |  |  |
|  | Ever-drinkers | 110 089 |  | 10 893 (9.9%) |  |  |
| Exercise frequency | |  |  |  |  | <0.0001 |
|  | 7 days/week | 236 257 |  | 13 120 (5.6%) |  |  |
|  | 1-6 days/week | 29 873 |  | 1 946 (6.5%) |  |  |
|  | <1day/week | 61 804 |  | 3 823 (6.2%) |  |  |
|  | No exercise | 1 559 776 |  | 134 760 (8.6%) |  |  |
| Dietary pattern | |  |  |  |  | <0.0001 |
|  | Omnivore diet | 1 703 369 |  | 135 650 (8.0%) |  |  |
|  | Plant-heavy diet | 122 280 |  | 12 176 (10.0%) |  |  |
|  | Meat-heavy diet | 62 061 |  | 5 823 (9.4%) |  |  |
| Weight group | |  |  |  |  | <0.0001 |
|  | Normal weight | 973 237 |  | 65 564 (6.7%) |  |  |
|  | Overweight or obesity | 914 473 |  | 88 085 (9.6%) |  |  |
| Diabetes | |  |  |  |  | <0.0001 |
|  | No | 1 805 071 |  | 140 062 (7.8%) |  |  |
|  | Yes | 82 639 |  | 13 587 (16.4%) |  |  |
| Hypertension | |  |  |  |  | <0.0001 |
|  | No | 1 632 674 |  | 101 870 (6.2%) |  |  |
|  | Yes | 255 036 |  | 51 779 (20.3%) |  |  |
| Hypercholesterolemia | |  |  |  |  | <0.0001 |
|  | No | 1 495 620 |  | 112 842 (7.5%) |  |  |
|  | Yes | 392 090 |  | 40 807 (10.4%) |  |  |
| Hypertriglyceridemia | |  |  |  |  | <0.0001 |
|  | No | 1 409 118 |  | 105 864 (7.5%) |  |  |
|  | Yes | 478 592 |  | 47 785 (10.0%) |  |  |

**Table S2: Sample sizes and prevalence of CVD in the study area by county**

| **County name** | **Participants (n)** | | **Number of cases** | | **Prevalence (%)** | |
| --- | --- | --- | --- | --- | --- | --- |
| Yingjisha County | 148 828 |  | 4 799 |  | 3.22 |  |
| Shule County | 54 907 |  | 1 954 |  | 3.56 |  |
| Jiashi County | 192 432 |  | 8 782 |  | 4.56 |  |
| Yopurga County | 88 835 |  | 4 871 |  | 5.48 |  |
| Tashkurgan Tajik County | 7 167 |  | 415 |  | 5.79 |  |
| Shufu County | 125 758 |  | 7 473 |  | 5.94 |  |
| Shache County | 425 882 |  | 27 296 |  | 6.41 |  |
| Makit County | 115 492 |  | 7 624 |  | 6.60 |  |
| Yecheng County | 305 025 |  | 21 181 |  | 6.94 |  |
| Bachu County | 145 145 |  | 16 127 |  | 11.11 |  |
| Zepu County | 114 277 |  | 20 677 |  | 18.09 |  |
| Kashi City | 163 962 |  | 32 450 |  | 19.80 |  |

**Table S3: Associations between CVD and candidate factors stratified by sex and ethnicity**

|  | | **OR (95% CI)** |  |  |  |  | |  |  |  |  | |  | | |  | |  | |  |
| --- | --- | --- | --- | --- | --- | --- | --- | --- | --- | --- | --- | --- | --- | --- | --- | --- | --- | --- | --- | --- |
|  | | **Sex-specific** | | | | |  | **Ethnicity-specific** | | | | | | |  | | | | |  |
|  | | **Man** |  |  | **Woman** | ***p* value** | |  | **Han** |  | | **Uyghur** | | | ***p* value** | |  | | |  |
| **Factors** | | **(n=923 655)** |  |  | **(n=964 055)** |  |  |  | **(n=56 078)** |  |  | **(n=1 811 752)** | | |  |  |  | | |  |
| Sex | |  |  |  |  |  | |  |  |  | |  | | |  | |  | | |  |
|  | Man | — —    ------------------------- | | | |  | |  | 1(Reference) |  | |  | | 1(Reference) |  | |  | | |  |
|  | Woman | — — | | | |  | |  | 1.33 (1.23-1.43) |  | |  | | 1.15 (1.13-1.16) | 0.07 | |  | | |  |
| Age(year) | |  |  |  |  |  | |  |  |  | |  | |  |  | |  | | |  |
|  | 18-45 | 1(Reference) |  |  | 1(Reference) |  | |  | 1(Reference) |  | |  | | 1(Reference) |  | |  | | |  |
|  | 45-65 | 1.93 (1.89-1.96) |  |  | 2.05 (2.01-2.08) | 0.02 | |  | 2.31 (2.14-2.50) |  | |  | | 1.99 (1.97-2.02) | 0.09 | |  | | |  |
|  | ≥65 | 3.07 (3.00-3.15) |  |  | 2.87 (2.80-2.94) | 0.04 | |  | 3.98 (3.61-4.40) |  | |  | | 2.96 (2.91-3.01) | 0.01 | |  | | |  |
| Ethnicity | |  |  |  |  |  | |  |  |  | |  | |  |  | |  | | |  |
|  | Han nationality | 1(Reference) |  |  | 1(Reference) |  | |  |  |  | |  | |  |  | |  | | |  |
|  | Uyghur nationality | 1.07 (1.02-1.12) |  |  | 1.00 (0.96-1.04) | 0.30 | |  | — — | | | | | |  | |  | | |  |
|  | Other | 1.16 (1.07-1.26) |  |  | 1.02 (0.94-1.11) | 0.29 | |  | — — | | | | | |  | |  | | |  |
| Residential setting | |  |  |  |  |  | |  |  |  | |  | |  |  | |  | | |  |
|  | Rural | 1(Reference) |  |  | 1(Reference) |  | |  | 1(Reference) |  | |  | | 1(Reference) |  | |  | | |  |
|  | Urban | 1.07 (1.05-1.09) |  |  | 1.00 (0.99-1.02) | 0.02 | |  | 0.81 (0.75-0.87) |  | |  | | 1.04 (1.03-1.06) | <0.0001 | |  | | |  |
| Economic status | |  |  |  |  |  | |  |  |  | |  | |  |  | |  | | |  |
|  | Poor household | 1(Reference) |  |  | 1(Reference) |  | |  | 1(Reference) |  | |  | | 1(Reference) |  | |  | | |  |
|  | Non-poor household | 1.06 (1.04-1.08) |  |  | 1.05 (1.04-1.07) | 0.88 | |  | 0.99 (0.85-1.16) |  | |  | | 1.05 (1.04-1.06) | 0.72 | |  | | |  |
| Education level | |  |  |  |  |  | |  |  |  | |  | |  |  | |  | | |  |
|  | Years of schooling >9  years | 1(Reference) |  |  | 1(Reference) |  | |  | 1(Reference) |  | |  | | 1(Reference) |  | |  | | |  |
|  | Years of schooling ≤9 | 1.13 (1.10-1.16) |  |  | 1.32 (1.29-1.36) | <0.0001 | |  | 1.16 (1.07-1.26) |  | |  | | 1.21 (1.19-1.24) | 0.64 | |  | | |  |
| Occupation | |  |  |  |  |  | |  |  |  | |  | |  |  | |  | | |  |
|  | Unemployed | 1(Reference) |  |  | 1(Reference) |  | |  | 1(Reference) |  | |  | | 1(Reference) |  | |  | | |  |
|  | Worker | 3.14 (3.00-3.29) |  |  | 2.61 (2.51-2.71) | 0.01 | |  | 1.30 (1.15-1.47) |  | |  | | 2.95 (2.86-3.04) | <0.0001 | |  | | |  |
|  | Farmer | 0.82 (0.78-0.85) |  |  | 0.77 (0.75-0.80) | 0.31 | |  | 0.93 (0.85-1.03) |  | |  | | 0.79 (0.77-0.82) | 0.16 | |  | | |  |
|  | Office clerk | 0.83 (0.78-0.88) |  |  | 0.78 (0.74-0.83) | 0.52 | |  | 0.66 (0.59-0.75) |  | |  | | 0.81 (0.77-0.84) | 0.13 | |  | | |  |
|  | Other | 0.65 (0.61-0.68) |  |  | 0.61 (0.59-0.64) | 0.41 | |  | 0.56 (0.50-0.62) |  | |  | | 0.63 (0.61-0.65) | 0.27 | |  | | |  |
| Smoking status | |  |  |  |  |  | |  |  |  | |  | |  |  | |  | | |  |
|  | Non- smokers | 1(Reference) |  |  | 1(Reference) |  | |  | 1(Reference) |  | |  | | 1(Reference) |  | |  | | |  |
|  | Current smokers | 1.18 (1.15-1.21) |  |  | 1.01 (0.85-1.18) | 0.32 | |  | 1.32 (1.19-1.46) |  | |  | | 1.19 (1.16-1.22) | 0.39 | |  | | |  |
|  | Former smokers | 1.3 0(1.25-1.35) |  |  | 1.34 (1.03-1.72) | 0.91 | |  | 1.45 (1.21-1.72) |  | |  | | 1.29 (1.24-1.34) | 0.57 | |  | | |  |
| Alcohol use | |  |  |  |  |  | |  |  |  | |  | |  |  | |  | | |  |
|  | Non-drinkers | 1(Reference) |  |  | 1(Reference) |  | |  | 1(Reference) |  | |  | | 1(Reference) |  | |  | | |  |
|  | Ever-drinkers | 1.27 (1.24-1.30) |  |  | 1.34 (1.15-1.55) | 0.75 | |  | 1.50(1.36-1.65) |  | |  | | 1.25 (1.22-1.29) | 0.12 | |  | | |  |
| Exercise frequency | |  |  |  |  |  | |  |  |  |  | | |  |  | | | |  | |
|  | 7 days/week | 1(Reference) |  |  | 1(Reference) |  | |  | 1(Reference) |  |  | | | 1(Reference) |  | | | |  | |
|  | 1-6 days/week | 1.11 (1.03-1.20) |  |  | 1.22 (1.13-1.30) | 0.40 | |  | 0.93 (0.76-1.14) |  |  | | | 1.18 (1.12-1.24) | 0.27 | | | |  | |
|  | <1day/week | 1.06 (1.01-1.12) |  |  | 1.07 (1.01-1.12) | 0.99 | |  | 0.80 (0.69-0.92) |  |  | | | 1.08 (1.04-1.12) | 0.03 | | | |  | |
|  | No exercise | 1.59 (1.55-1.64) |  |  | 1.62 (1.58-1.67) | 0.67 | |  | 0.88 (0.81-0.95) |  |  | | | 1.65 (1.62-1.68) | <0.0001 | | | |  | |
| Dietary pattern | |  |  |  |  |  | |  |  |  |  | | |  |  | | | |  | |
|  | Omnivore diet | 1(Reference) |  |  | 1(Reference) |  | |  | 1(Reference) |  |  | | | 1(Reference) |  | | | |  | |
|  | Plant-heavy diet | 1.61 (1.57-1.66) |  |  | 1.14 (1.11-1.18) | <0.0001 | |  | 1.40 (1.25-1.58) |  |  | | | 1.36 (1.33-1.38) | 0.78 | | | |  | |
|  | Meat-heavy diet | 1.23 (1.18-1.28) |  |  | 1.24 (1.19-1.29) | 0.93 | |  | 1.28 (1.07-1.53) |  |  | | | 1.24 (1.21-1.28) | 0.87 | | | |  | |
| Weight group | |  |  |  |  |  | |  |  |  |  | | |  |  | | | |  | |
|  | Normal weight ^a^ | 1(Reference) |  |  | 1(Reference) |  | |  | 1(Reference) |  |  | | | 1(Reference) |  | | | |  | |
|  | Overweight or obesity | 1.00 (0.98-1.01) |  |  | 1.16 (1.14-1.18) | <0.0001 | |  | 1.13 (1.06-1.20) |  |  | | | 1.08 (1.06-1.09) | 0.50 | | | |  | |
| Diabetes ^b^ | |  |  |  |  |  | |  |  |  |  | | |  |  | | | |  | |
|  | No | 1(Reference) |  |  | 1(Reference) |  | |  | 1(Reference) |  |  | | | 1(Reference) |  | | | |  | |
|  | Yes | 1.32 (1.28-1.36) |  |  | 1.40 (1.36-1.44) | 0.17 | |  | 1.25 (1.13-1.38) |  |  | | | 1.37 (1.34-1.40) | 0.36 | | | |  | |
| Hypertension ^c^ | |  |  |  |  |  | |  |  |  |  | | |  |  | | | |  | |
|  | No | 1(Reference) |  |  | 1(Reference) |  | |  | 1(Reference) |  |  | | | 1(Reference) |  | | | |  | |
|  | Yes | 2.39 (2.34-2.43) |  |  | 2.79 (2.74-2.84) | <0.0001 | |  | 2.92 (2.73-3.12) |  |  | | | 2.59 (2.56-2.63) | 0.12 | | | |  | |
| Hypercholesterolemia ^d^ | |  |  |  |  |  | |  |  |  |  | | |  |  | | | |  | |
|  | No | 1(Reference) |  |  | 1(Reference) |  | |  | 1(Reference) |  |  | | | 1(Reference) |  | | | |  | |
|  | Yes | 1.07 (1.05-1.09) |  |  | 1.07 (1.06-1.09) | 0.89 | |  | 1.13 (1.05-1.21) |  |  | | | 1.08 (1.06-1.09) | 0.53 | | | |  | |
| Hypertriglyceridemia ^e^ | |  |  |  |  |  | |  |  |  |  | | |  |  | | | |  | |
|  | No | 1(Reference) |  |  | 1(Reference) |  | |  | 1(Reference) |  |  | | | 1(Reference) |  | | | |  | |
|  | Yes | 1.03 (1.01-1.05) |  |  | 1.10 (1.08-1.12) | 0.01 | |  | 1.00 (0.94-1.08) |  |  | | | 1.07 (1.05-1.08) | 0.38 | | | |  | |

Abbreviation: CI, confidence interval; OR, odds ratio.

**Table S4: Area under the receiver operating characteristic (ROC) curve and area under the precision-recall (PR) curve for RF, RFs, and XGBoost algorithms**

| Classifier | The area under the ROC curve | The area under the PR curve |
| --- | --- | --- |
| Random Forest (RF) | 0.723 | 0.226 |
| Random Ferns (RFs) | 0.701 | 0.193 |
| Extreme Gradient Boosting (XGBoost) | 0.723 | 0.223 |

**Table S5:** **AUC values in stepwise selection procedure using RF algorithm**

| Number of factors | | AUC |
| --- | --- | --- |
| 1 |  | 0.644 |
| 2 |  | 0.672 |
| 3 |  | 0.701 |
| 4 |  | 0.710 |
| 5 |  | 0.715 |
| 6 |  | 0.715 |
| 7 |  | 0.716 |
| 8 |  | 0.717 |
| 9 |  | 0.721 |
| 10 |  | 0.721 |
| 11 |  | 0.722 |
| 12 |  | 0.723 |
| 13 |  | 0.723 |
| 14 |  | 0.723 |
| 15 |  | 0.723 |
| 16 |  | 0.723 |

**Table S6: Factor rankings for CVD computed by RF algorithm by sex, ethnicity, education level, economic status, and residential setting**

| **Sub-group** | | **Importance ranking** | **OOB error** |
| --- | --- | --- | --- |
| **Sex** | Man | 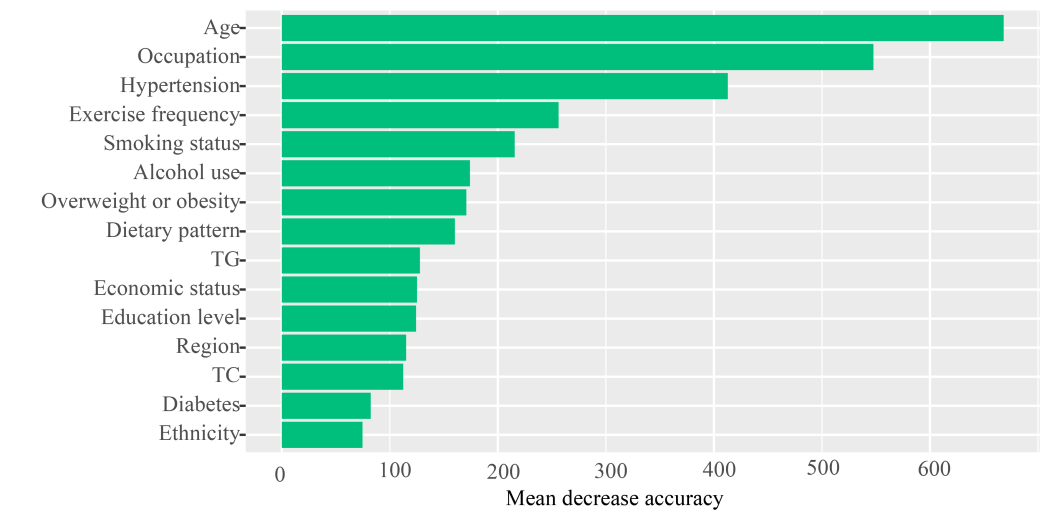 | 0.199 |
|  | Woman | 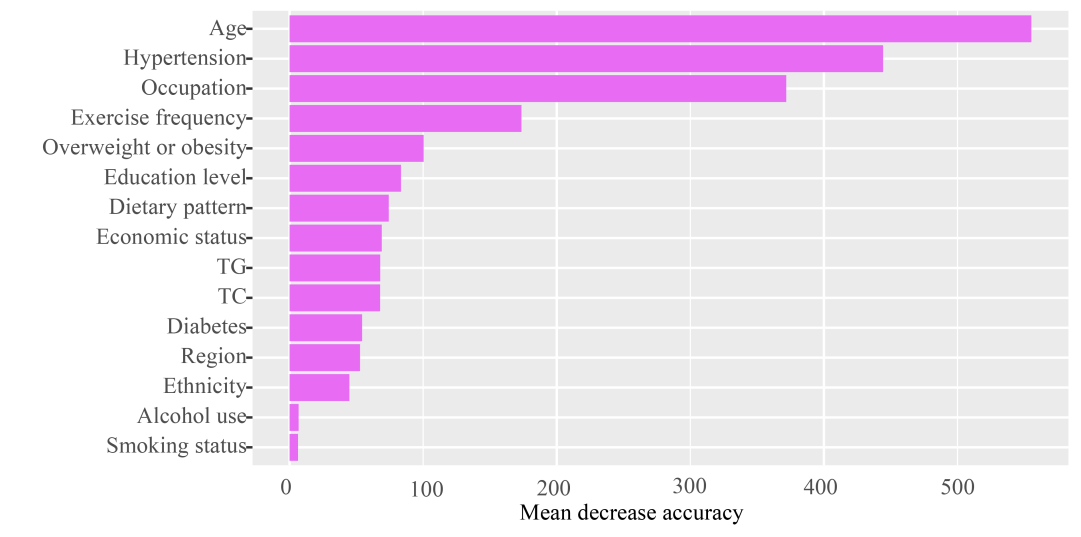 | 0.199 |
| **Ethnicity** | Han | 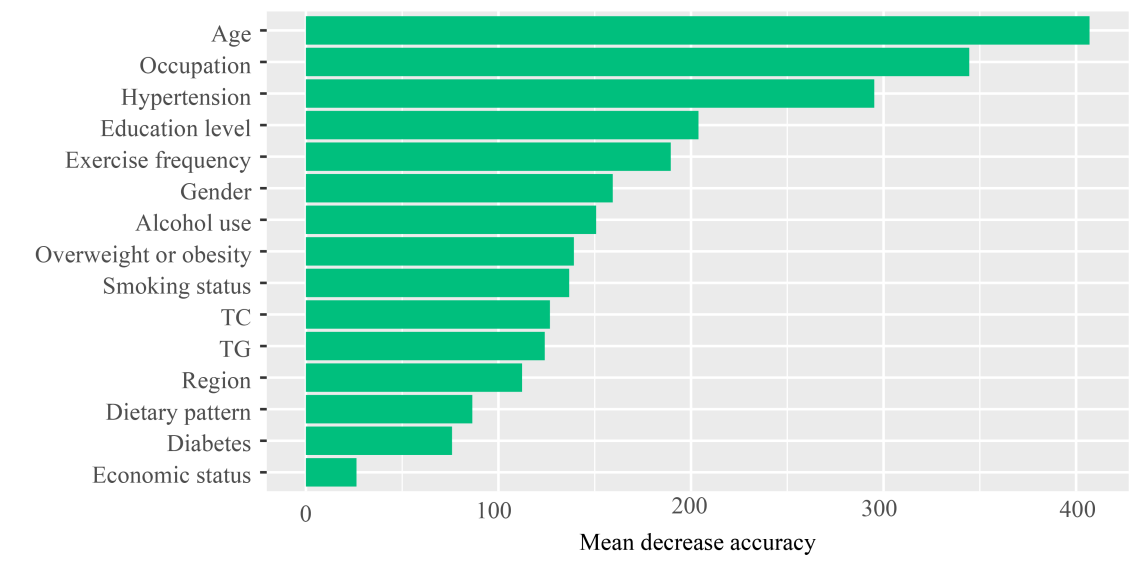 | 0.174 |
|  | Uyghur | 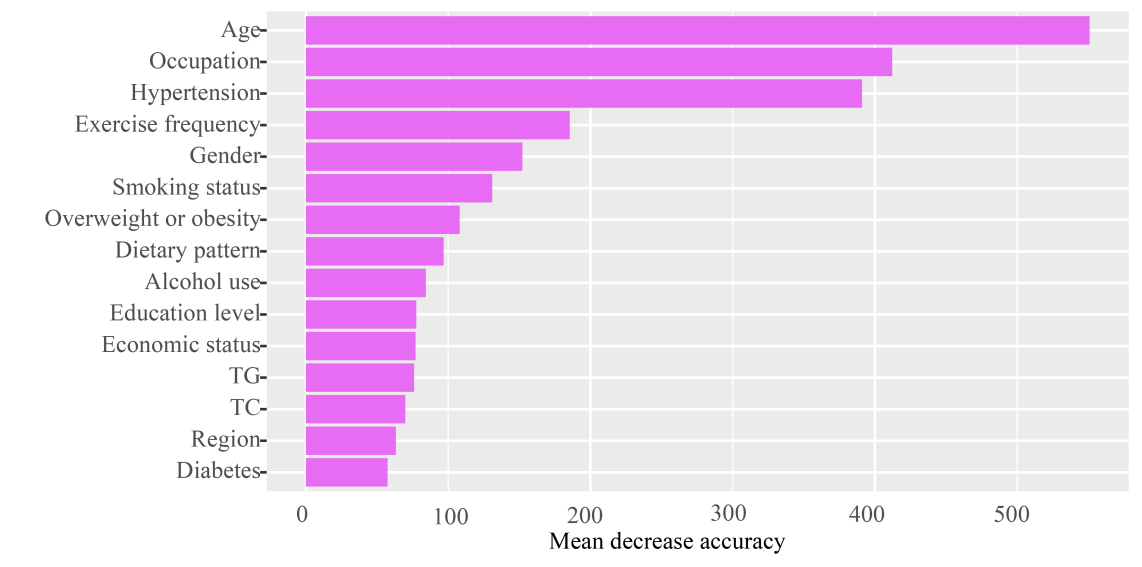 | 0.200 |
| **Education level** | Schooling  >  9 years | 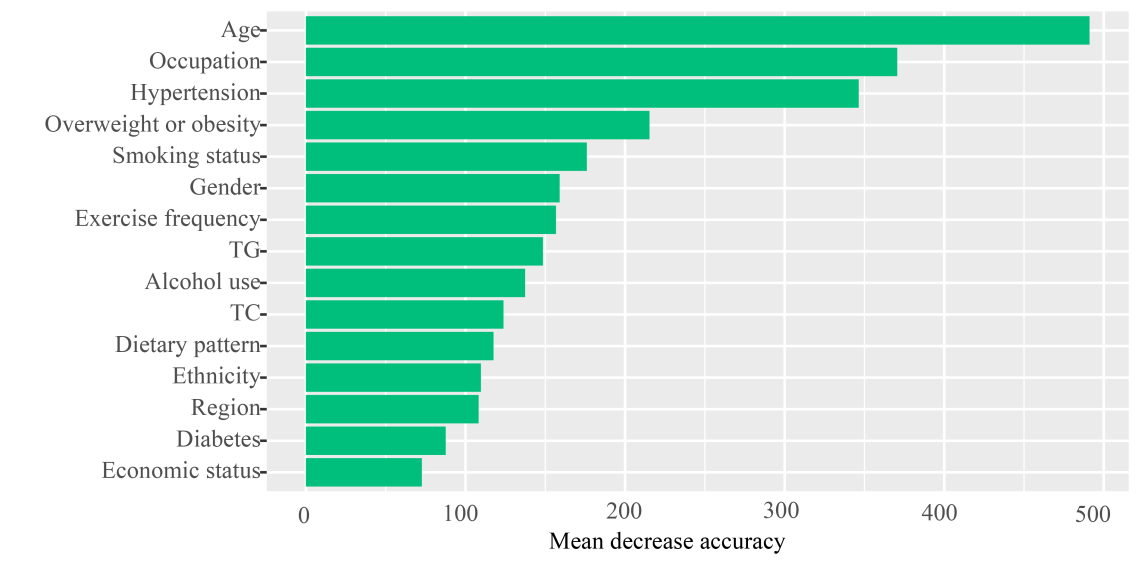 | 0.200 |
|  | Schooling  ≤  9 years | 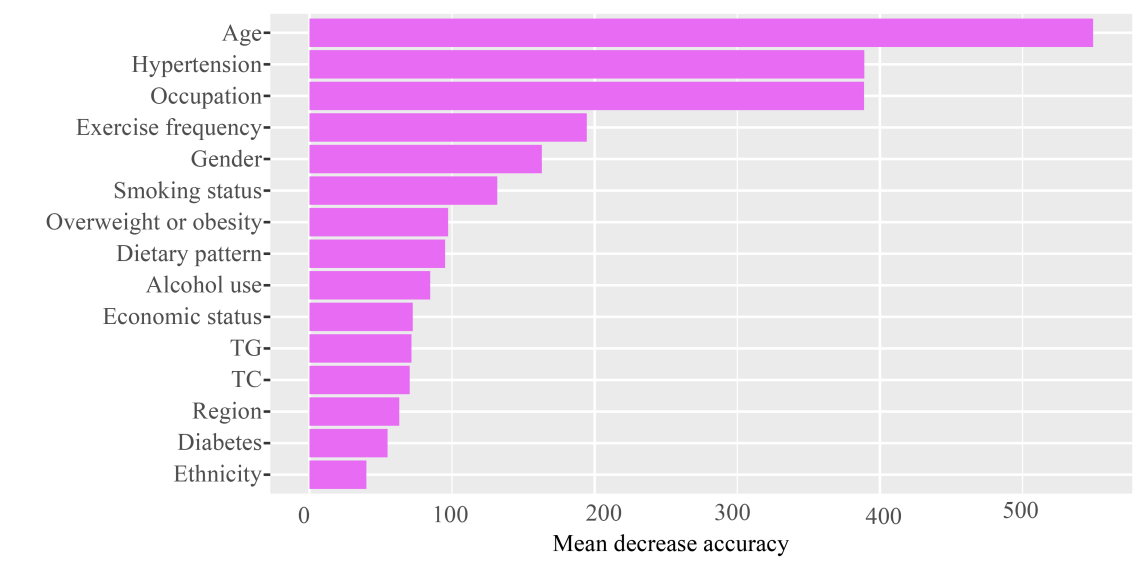 | 0.185 |
| **Economic status** | Poor household | 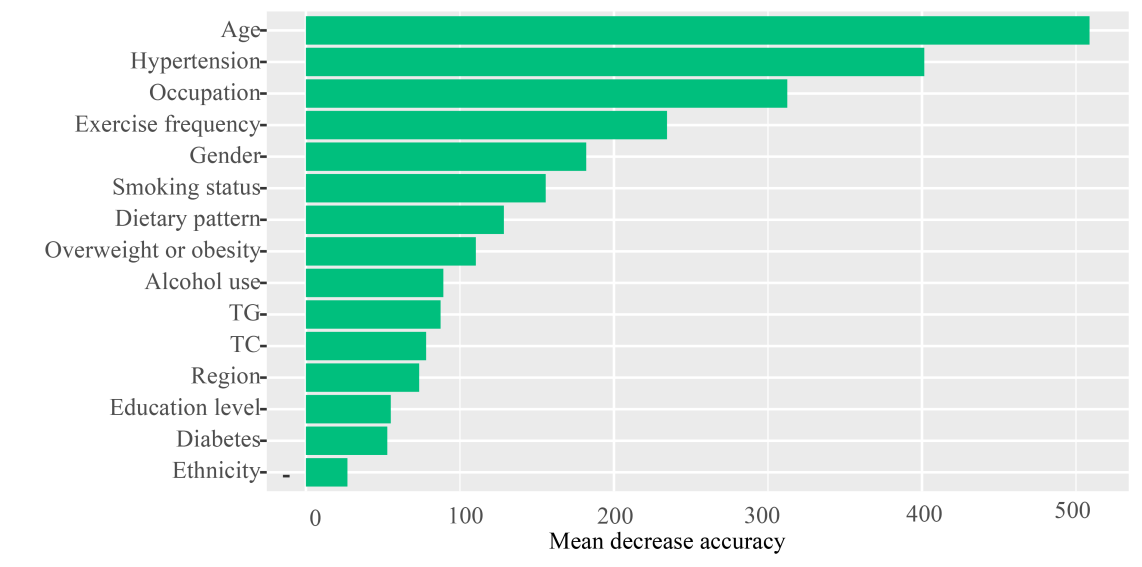 | 0.197 |
|  | Non-poor household | 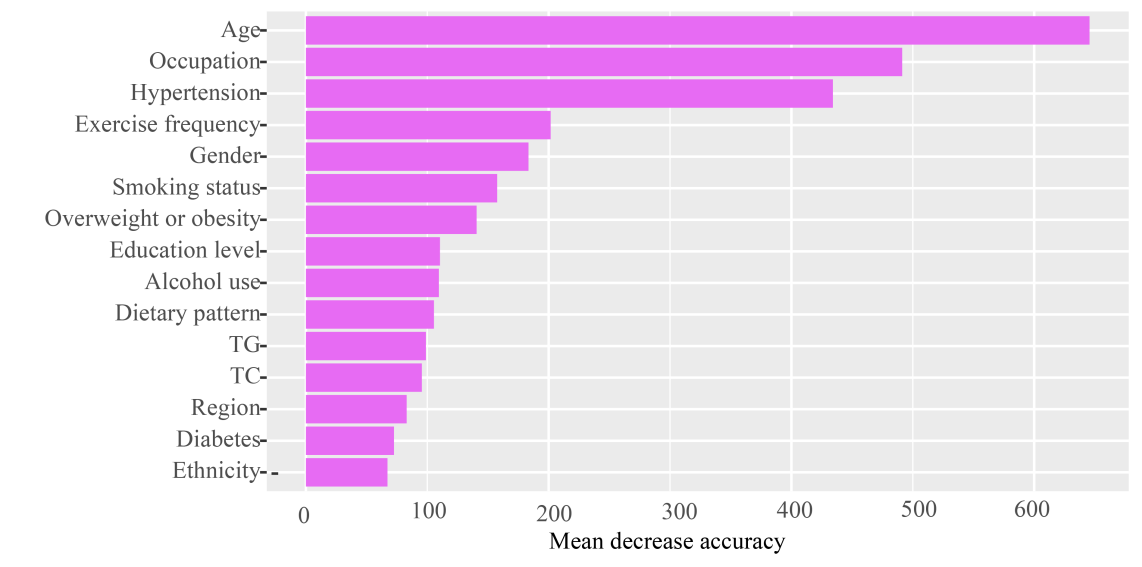 | 0.204 |
| **Residential setting** | Rural | 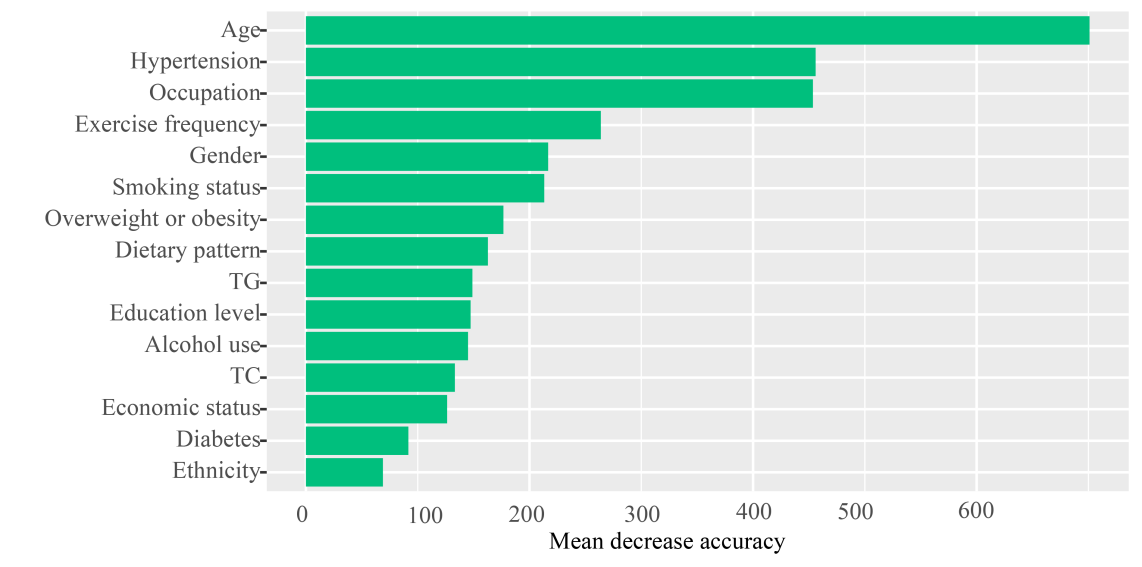 | 0.201 |
|  | Urban | 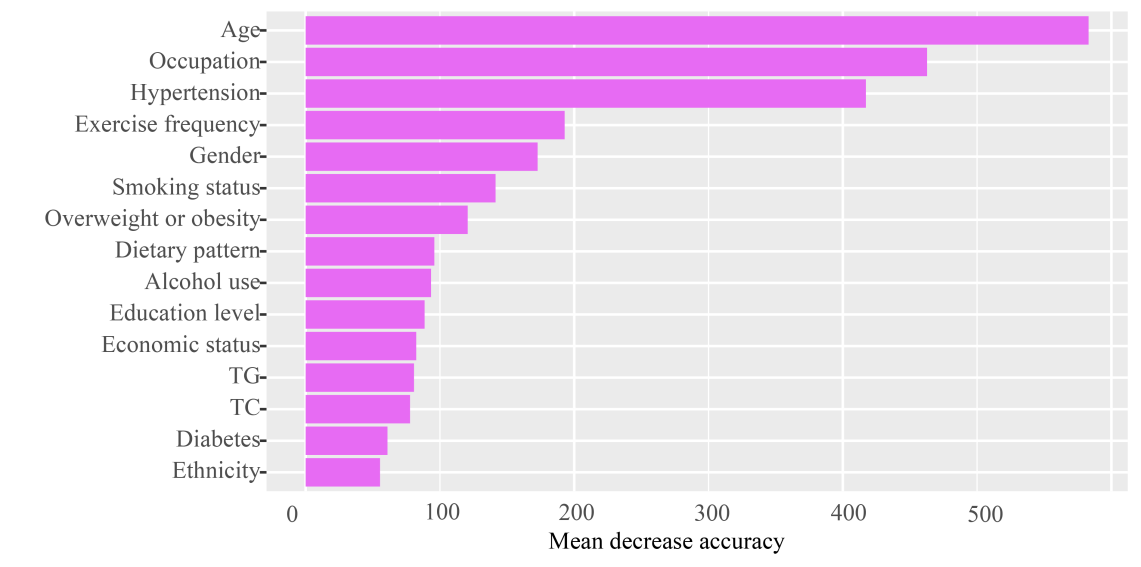 | 0.163 |

Note: OOB error means the out-of-bag error, an indicator evaluating the performance of Random Forest models. The smaller the OOB, the better the model performance.

**References**

1. Chawla NV, Lazarevic A, Hall LO, Bowyer KW. SMOTEBoost: improving prediction of the minority class in boosting. Berlin, Germany: Springer, 2003.

2. Kursa MB, Rudnicki WR. Feature selection with the Boruta package. *J. Stat. Softw* 2010; **36**(11): 1-13.

3. Hong Y, Kwong S, Chang YC, Ren QS. Consensus unsupervised feature ranking from multiple views. *Pattern Recognit Lett* 2008; **29**(5): 595-602.

4. Bosch A, Zisserman A, Munoz X, Ieee, editors. Image classification using random forests and ferns. *ICCV IEEE* 2007; published online Dec 26. DOI:10.1109/ICCV.2007.4409066.

5. Unterhuber M, Kresoja KP, Rommel KP, Besler C, Baragetti A, Klöting N, et al. Proteomics-enabled deep learning machine algorithms can enhance prediction of mortality. *J Am Coll Cardiol* 2021; **78**(16): 1621-31.

6. Strobl C, Boulesteix AL, Zeileis A, Hothorn T. Bias in random forest variable importance measures: illustrations, sources and a solution. *BMC Bioinform* 2007; **8**: 25.

7. Kursa MB. rFerns: an implementation of the Random Ferns method for general purpose machine learning. *J. Stat. Softw* 2014; **61**(10): 1-13.

8. Friedman J, Hastie T, Tibshirani R. The elements of statistical learning. Berlin, Germany: Springer, 2001.

9. Bradley AP. The use of the area under the ROC curve in the evaluation of machine learning algorithms. *Pattern Recognit* 1997; **30**(7): 1145-60.

10. Saito T, Rehmsmeier M. The precision-recall plot is more informative than the ROC plot when evaluating binary classifiers on imbalanced datasets. *PLos One* 2015; **10**(3): e0118432.

11. Krittanawong C, Virk HUH, Bangalore S, Wang Z, Johnson KW, Pinotti R, et al. Machine learning prediction in cardiovascular diseases: a meta-analysis. *Sci Rep* 2020; **10**(1): 16057.

12. Government of the People's Republic of China. Xinjiang: 2017 national health checkup work completed. http://www.gov.cn/xinwen/2017-11/02/content_5236389.htm (accessed Feb 22, 2022).

13. Zhu H, Zhou H, Chen X, Ma J. Analysis on the characteristics of groundwater resources in Kashgar prefecture, Xinjiang. *Arid Zone Res* 2005; **02**: 152-6 (in Chinese).

14. Cai J, Huang X, Zhang Y, Xiao H. Assessing the dietary nutritional status of Uygur adult residents of Kashi region in Xinjiang using diet balance index. *Weisheng Yanjiu* 2018; **47**(4): 562-76 (in Chinese).

15. Xinjiang Uyghur Autonomous Region Health Commission. National health checkup work implementation plan. http://www.xinjiang.gov.cn/xinjiang/fgwjx/201909/d4be51c88f6e4b1cab22dd764c88b3ef.shtml (accessed Feb 22, 2022).

16. National Health and Family Planning Commission of the People’s Republic of China. National Basic Public Health Service Specifications (Third Edition). http://wjw.beijing.gov.cn/wjwh/ztzl/ggwsfw/201912/P020191217743891499573.pdf (accessed Feb 22, 2022).
